# Hybrid Functional DFTB Parametrizations for Modeling Organic Photovoltaic Systems

**DOI:** 10.1021/acs.jctc.5c00232

**Published:** 2025-05-08

**Authors:** Wenbo Sun, Tammo van der Heide, Van-Quan Vuong, Thomas Frauenheim, Michael A. Sentef, Bálint Aradi, Carlos R. Lien-Medrano

**Affiliations:** † Institute for Theoretical Physics and Bremen Center for Computational Materials Science, 530484 University of Bremen, 28359 Bremen, Germany; ‡ Institute for Physical Chemistry, 150232 Karlsruhe Institute of Technology, 76131 Karlsruhe, Germany; § School of Science, Constructor University, Campus Ring 1, 28759 Bremen, Germany; ∥ Institute for Advanced Study, 9168 Chengdu University, Chengdu 610106, P. R. China

## Abstract

Density functional tight binding (DFTB) is a quantum chemical simulation method based on an approximate density functional theory (DFT), known for its low computational cost and comparable accuracy to DFT. For several years, the application of DFTB in organic photovoltaics (OPV) has been limited by the absence of an appropriate set of parameters that adequately account for the relevant elements and necessary corrections. Here we have developed new parametrizations using hybrid functionals, including B3LYP and CAM-B3LYP, for OPV applications within the DFTB method in order to overcome the self-interaction error present in DFT functionals lacking long-range correction. These parametrizations encompass electronic and repulsive parameters for the elements H, C, N, O, F, S, and Cl. A Bayesian optimization approach was employed to optimize the free atom eigenenergies of unoccupied shells. The effectiveness of these new parametrizations was evaluated by a data set of 12 OPV donor and acceptor molecules, showing consistent performance when compared with their corresponding DFT references. Frontier molecular orbitals and optimized geometries were examined to evaluate the performance of the new parametrizations in predicting ground-state properties. Furthermore, the excited-state properties of monomers and dimers were investigated by means of real-time time-dependent DFTB (real-time TD-DFTB). The appearance of charge-transfer (CT) excitations in the dimers was observed, and the influence of alkyl side-chains on the photoinduced CT process was explored. This work paves the way for studying ground- and excited-state properties, including band alignments and CT mechanisms at donor–acceptor interfaces, in realistic OPV systems.

## Graphical abstract

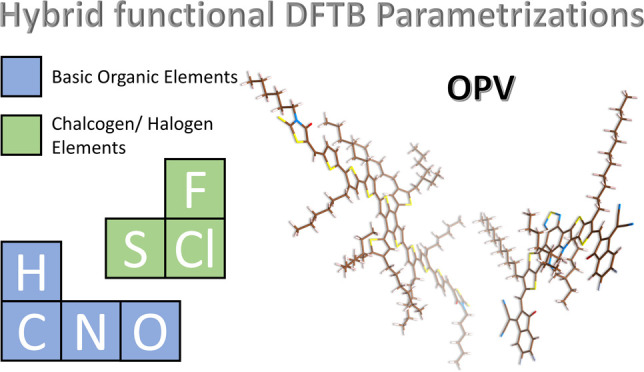

## 1 Introduction

In recent years, the field of organic photovoltaics (OPV) has attracted widespread attention due to its potential for low cost, high power conversion efficiency (PCE), and flexibility. It has witnessed significant progress, marked by the synthesis of novel donor and nonfullerene acceptor molecules, with power conversion efficiencies (PCEs) approaching 20%.^1–5^ A bulk-heterojunction (BHJ) OPV device^6^ comprises four distinct layers arranged from bottom to top: the substrate, the anode electrode, the active layer (consisting of donor and acceptor materials), and the cathode electrode. In OPV devices, the operation involves three main processes:^7^ (i) light absorption, where photoexcitation in the active layer generates excitons; (ii) charge separation, which occurs at the donor–acceptor interface as excitons dissociate into free charge carriers; and (iii) charge transport, where the separated electrons and holes are driven toward the respective electrodes. Finally, electrodes collect the charges and transfer them to the external circuit for electricity generation. To gain deeper insights into the mechanisms of OPV and to predict the electronic properties of molecules, theoretical computational methods are commonly employed. For example, determining the geometries of novel molecules typically necessitates the assistance of computational methods.^8^ Besides, predicting frontier molecular orbital levels is essential to ensure compatibility between donor and acceptor materials.^9,10^ Moreover, properties such as absorption spectrum,^11^ exciton binding energy,^12^ electrostatic potential,^13^ and carrier mobility^10,14–16^ can be predicted and the property-structure relationship can be studied^17^ by theoretical computations, accelerating the process of material design.

To study intermolecular interfaces and molecular packing configurations, models comprising dimers or aggregates of donor–acceptor molecules are required.^18–21^ For example, Wei et al. demonstrated the critical role of three-dimensional molecular packing of donor–acceptor molecules in reducing the exciton binding energy, thereby enhancing open-circuit voltages.^22^ However, density functional theory (DFT) calculations pose a challenge when treating such large systems, owing to the computational resources required. As an alternative, the density functional tight binding (DFTB) method,^23^ a direct approximation to Kohn–Sham DFT, is orders of magnitude faster than DFT methods, enabling efficient calculations of large systems. Notably, DFTB has been employed to investigate charge-transfer (CT) processes at the donor–acceptor interfaces in OPV. By applying DFTB and Ehrenfest molecular dynamics to a P3HT (donor)/PCBM (acceptor) system, Chen et al. explored the role of vibronic coupling in CT across donor–acceptor interfaces.^24^ Subsequently, they investigated various stages in a dimer system, including electron–hole pair formation in the donor material, the dissociation at the donor–acceptor interface, and electron and hole transport for photocurrent generation.^25^ Additionally, Nakai and Uratani employed a model consisting of 2 P3HT decamers and 16 PCBM molecules to study the electron-transfer pathway and its driving force using DFTB and the patchwork-approximation (PA)-based Ehrenfest method.^26^

The accuracy and performance of DFTB methods are highly dependent on the quality of parametrization and the functionals used during parametrization. The availability of DFTB parameters for a wide range of chemical elements, ideally covering the entire periodic table, is important for broad applications using DFTB.^27^ Typically, widely employed parameter sets^28,29^ are based on the semilocal Perdew–Burke–Ernzerhof (PBE) functional.^30^ However, the PBE functional suffers from an error arising from residual self-interaction, resulting in an incorrect behavior of the Kohn–Sham potential in the long-range as well as an overdelocalization of the electron density.^31^ In particular, these inaccuracies lead to the underestimation of CT excited states,^32^ impacting properties relevant to OPV applications.

To enhance the description of excited state properties within DFTB, incorporating long-range corrected hybrid functionals (LC-DFTB), which partition the electron–electron interaction into short- and long-range contributions, deals with the delocalization problem to a great extent. On one hand, Niehaus and Sala developed the methodological extension of the DFTB formalism to range-separated purely long-range corrected hybrid functionals for molecules,^33^ which has later been implemented and benchmarked by Lutsker and co-workers.^34^ Subsequently, the extension of the time-dependent formulation to LC-DFTB has enabled the investigation of excited-state properties.^35,36^ Recent developments also enabled LC-DFTB calculations of ground-state properties for periodic systems.^37^ On the other hand, Elstner et al. parametrized the *ob2–1–1* set^38^ for LC-DFTB using the LC-BNL functional,^39^ which includes the chemical elements H, C, N, and O. Based on an extension of the *ob2–1–1* parameter set, Varella et al. studied the effect of polymer–fullerene interface configurations, specifically face-on and edge-on orientations, on CT excitons using LC-DFTB.^40^ It was demonstrated that if the acceptor molecule is located in an edge-on orientation with respect to the donor molecule, the density of cold CT excitons is higher, leading to a more efficient charge separation.

In this work, different functionals, including B3LYP^41^ and CAM-B3LYP,^42^ were parametrized for DFTB. Additionally, a parameter set using the LC-BNL functional was also developed based on a previous work,^40^ where the chemical element S was reparametrized and subsequently extended to include the elements F and Cl, only for comparative purposes. Note that all range-separated parametrizations referenced herein, as well as those generated as part of this work, employ a range-separation function of Yukawa type. We omit to indicate this deviation from an error-function type partitioning for brevity and e.g., refer to LCY-BNL (CAMY-B3LYP) as LC-BNL (CAM-B3LYP) throughout. For the B3LYP and CAM-B3LYP functionals, the chemical elements H, C, N, O, F, S, and Cl were parametrized from scratch. This extension of parameters broadens the applicability of LC-DFTB to investigating a more diverse range of molecules relevant to OPV, such as the recently designed nonfullerene acceptor Y6 and its derivatives,^8,43^ the ITIC-family of acceptors,^44^ as well as small-molecule BDT-core based donors,^45,46^ along with their respective dimers. The performance of different functionals has been compared for both ground- and excited-state properties. A donor–acceptor dimer system has been investigated using electron dynamics on the LC-DFTB level of theory to characterize the CT excitation. Additionally, the computational efficiency of DFTB allowed us to study the influence of side-chains on CT excitations.

## 2 Methods

### 2.1 LC-DFTB Theory

Based on the Coulomb-attenuating method (CAM),^47^ the electron–electron interaction is partitioned into short- and long-range contributions, governed by three parameters α, β, and ω. The parameter α determines the fraction of global Hartree–Fock (HF) type exchange over the entire range, while β specifies the fraction of the HF exchange in the long-range. Consequently, the DFT component across the entire range is determined by the factor 1–(α + β). Additionally, ω is the range-separation parameter, modulating the transition from DFT to HF exchange. The Coulomb operator is represented as a summation of short- and long-range components in eq 1

1 $$\frac{1}{r}=\frac{1-\left(\alpha +\beta \right)+\beta e^{-\omega r}}{r}+\frac{\alpha +\beta \left(1-e^{-\omega r}\right)}{r}$$

There are two types of hybrid functionals, i.e., the global hybrid functional B3LYP and the range-separated hybrid functionals CAM-B3LYP and LC-BNL, which have already been integrated into the DFTB methods within the *DFTB+* software package.^23^ The global hybrid functional B3LYP employs parameters specified in eq 1 with α = 0.2 and β = 0 to mix the exchange

2 $$E_{\mathrm{xc}}=\alpha E_{x}^{\mathrm{HF}}+\left(1-\alpha \right)E_{x}^{\mathrm{DFT}}+E_{c}^{\mathrm{DFT}}$$

where *E*_x_^HF^, *E*_x_^DFT^, and *E*_c_^DFT^ refer to the HF exchange, the semilocal DFT exchange, and the DFT correlation energy, respectively. Thus, the B3LYP functional incorporates 20% of exact HF exchange. In addition, the range-separated hybrid functional LC-BNL is defined by the parameters α = 0 and β = 1, yielding a purely long-range corrected functional. The CAM-B3LYP functional offers more flexibility due to the adjustable parameters α and β, allowing for the tuning of HF and DFT exchange contributions in the short- and long-range. With different combinations of α, β, and ω, the exchange-correlation energy of range-separated hybrid functionals can be expressed as

3 $$E_{\mathrm{xc}}=\alpha E_{x}^{\mathrm{HF}}+\left[1-\left(\alpha +\beta \right)\right]E_{x}^{\mathrm{DFT}}+\beta \left(E_{x,\mathrm{sr}}^{\omega ,\mathrm{DFT}}+E_{x,\mathrm{lr}}^{\omega ,\mathrm{HF}}\right)+E_{c}^{\mathrm{DFT}}$$

where *E*_x,sr_^ω,DFT^ and *E*_x,lr_^ω,HF^ stand for the short-range DFT exchange and the long-range HF exchange, respectively. The generalized Kohn–Sham (GKS) total energy is written as

4 $$E\left[\rho \right]=T\left[\rho \right]+E_{\mathrm{ext}}\left[\rho \right]+E_{H}\left[\rho \right]+E_{\mathrm{NN}}+E_{\mathrm{xc}}$$

where *T*[ρ], *E*_ext_[ρ], *E*_H_[ρ], *E*_NN_, and *E*_xc_ represent the kinetic energy, external potential, Hartree energy, nuclear-repulsion energy, and exchange-correlation term, respectively. Analogous to the expansion used in the standard DFTB formalism, the total energy of eq 4 is expanded around a reference density matrix up to the second order.

The DFTB method usually uses a minimal atomic basis set ϕ_μ_(*r*) to construct the molecular orbitals (MOs) Ψ_*i*_(*r*) through a linear combination of atomic orbitals (LCAO)

5 $$\Psi_{i}\left(r\right)=\sum_{\mu }c_{\mu ,i}\phi_{\mu }\left(r\right)$$

In order to obtain this basis set, first-principles hybrid DFT calculations are performed for pseudoatoms by solving the atomic GKS equations with an additional confinement potential $V_{\mathrm{conf}}\left(r\right)={\left(\frac{r}{r_{0}^{\mathrm{wf}}}\right)}^{n}$ , where *r*_0_^wf^ denotes the wave function compression radius for each element

6 $$\left[-\frac{1}{2}\nabla^{2}+V^{\mathrm{eff}}+{\left(\frac{r}{r_{0}^{\mathrm{wf}}}\right)}^{n}\right]\phi_{\mu }=\epsilon_{\mu }\phi_{\mu }$$

Similarly, another density compression radius *r*_0_^den^ is employed to determine the initial guess of atomic densities for neutral atoms. The atomic orbital density matrix *P*_μν_ is defined as

7 $$P_{\mu \nu }=2\sum_{i=1}^{N/2}n_{i}c_{\mu ,i}c_{\nu ,i}$$

where *n*_*i*_ is the occupancy of spin–orbitals. In the DFTB method, the total energy is expressed as an expansion using a reference density and its fluctuation to represent the ground state density, given by *P* = *P*_0_ + Δ*P*. The reference density matrix *P*_0_ is the superposition of the corresponding atomic density matrices of all atoms in the system ({*A*}): *P*_0_ = ∑_*A*_ *P*_*A*_. Thus, the total energy expression of LC-DFTB is written as

8 $$E=\sum_{\mu \nu }P_{\mu \nu }H_{\mu \nu }^{\left(0\right)}+E_{\mathrm{DFT}}^{\left(2\right)}+E_{\mathrm{HF}}^{\left(2\right)}+E_{\mathrm{rep}}$$

Using a two-center approximation, the zeroth-order Hamiltonian matrix elements *H*_μν_^(0)^ can be evaluated as

9 $$H_{\mu \nu }^{\left(0\right)}=\{\begin{array}{ll} \epsilon_{\mu }^{\mathrm{free}} & \mu =\nu \\ H_{\mu \nu }^{\left(0\right)}\left[P_{A}+P_{B}\right] & \mu \in A,\nu \in B\\ 0 & \mathrm{else} \end{array}$$

where ϵ_μ_^free^ are the eigenenergies of free atom orbitals obtained from first-principle calculations. The off-site matrix elements are constructed from a pretabulated table of integrals of high-symmetry orbital configurations as a function of the interatomic distance *R_AB_* between atoms *A* and *B*. The terms *E*_DFT_^(2)^ and *E*_HF_^(2)^ have been described in detail in previous works,^33,34,37^ aligning with the GKS total energy expression. In addition, the repulsive energy term *E*_rep_ is calculated using a two-center approximation with short-ranged pair-potentials:

10 $$E_{\mathrm{rep}}=\frac{1}{2}\sum_{A,B\neq A}V_{\mathrm{AB}}\left(R_{\mathrm{AB}}\right)$$

### 2.2 Parametrization for LC-DFTB

During the parametrization process, two types of parameters need to be determined: the electronic parameters and the repulsive parameters. The electronic parameters are obtained from GKS-DFT calculations of pseudoatoms, incorporating additional adjustable parameters, which were introduced in Section 2.1 as the (shell-resolved) wave function compression radii *r*_0_^wf^ and the density compression radius *r*_0_^den^. Additionally, the free atom eigenenergies ϵ_μ_^free^ of occupied shells are computed using GKS-DFT. As we have discussed in ref 48, the on-site energies of unoccupied shells are associated with unbound scattering-states and are ill-defined for the isolated neutral pseudoatom calculations. We, therefore, treat unoccupied on-site energies as free, exchange-correlation functional-dependent parameters. To represent the repulsive energy, we resort to the usual piecewise function composed of an exponential head, cubic splines in the bonding region, and a fifth-order polynomial for damping the repulsive contributions to zero when approaching the cutoff distance. The repulsive parameters are determined by fitting the total DFTB energy to GKS-DFT references generated for a data set of small molecules.

#### 2.2.1 Electronic Parameters and Bayesian Optimization

The electronic parameters were generated using the *SkProgs* package,^49^ which supports global hybrid functionals and range-separated hybrids. *SkProgs* employs a Slater-type orbital (STO) basis for atomic calculations, with expansion coefficients and exponents determined during the electronic parametrization process. During parametrization, it was observed that the range-separation parameter ω of range-separated hybrid functionals significantly influences the highest occupied molecular orbital (HOMO)–lowest unoccupied molecular orbital (LUMO) gaps of the target molecules. To ensure a reasonable comparison between experimental and computational results, ω = 0.1 was selected for the parametrization. This relatively small ω value, around 0.1, has also been suggested by other DFT studies on OPV molecules.^50–52^ For the same reason, nonstandard parameters α = 0.05 and β = 0.7 were chosen for the CAM-B3LYP functional. To distinguish our CAM-B3LYP-based parameter set from the original CAM-B3LYP functional with different range-separation parameters, we denote our parameter set as CAM-B3LYP′.

The compression radii *r*_0_^wf^ and *r*_0_^den^ for the elements H, C, N, and O were adopted from a previous study,^40^ which extended the original *ob2–1–1* set to include sulfur (S). However, we found that the wave function compression radius of the 3*d*-orbital of S was set to a small value (below 2 a_0_), leading to unphysical relaxed geometries (as detailed in the Supporting Information, Figure S1). Therefore, we reparametrized the sulfur parameters and extended the parameter set to include fluorine (F) and chlorine (Cl). The parameters used for electronic parametrization are summarized in Tables 1 and S1.

**Table 1:** Optimized Atomic Parameters Used to Generate Electronic Parametrization of the B3LYP and the CAM-B3LYP′ Functionals for Different Elements^a^

functional	parameters	H	C	N	O	F	S	Cl
B3LYP	power	2	2	2	2	2	4	9
	*r* ^den^	3.2	7.6	4.6	5.0	4.0	8.0	8.0
	*r*_sp_^wf^	3.2	3.8	2.3	2.5	2.8	4.0	4.0
	*r*_d_^wf^ (ϵ_μ_^free^)						4.5 (0.091)	3.5 (0.067)
CAM-B3LYP′	power	2	2	2	2	2	4	9
	*r* ^den^	3.2	7.6	4.6	5.0	4.0	8.0	9.0
	*r*_sp_^wf^	3.2	3.8	2.3	2.5	2.8	4.0	3.0
	*r*_d_^wf^ (ϵ_μ_^free^)						4.5 (0.124)	3.0 (0.171)

a Parameters are given in atomic units (*r* ^den^ [a_0_], *r* ^wf^ [a_0_], and ϵ_μ_^free^ [Ha]).

It is important to note that choosing the optimal compression for sulfur is a challenging problem in DFTB. As highlighted in a previous study,^53^ calculations using the *mio-1–1* parameter set yield artificial local minima in noncovalent interactions involving sulfur atoms. Our calculations (Figure S4) indicate that no such artifacts occur when using the extended *ob2–1–1* parameter set with its strongly compressed sulfur atom. Unfortunately, as already mentioned above, the strong compression leads to unphysical optimized geometries both for small molecules such as H_2_SO_4_ (as discussed in Figure S1), and larger systems such as Y6-family acceptors (see Figure S5). Apparently, there exists a clear trade-off in the choice of the sulfur *d*-orbital compression radius, which affects the balance between accurately describing interaction energies and achieving reliable optimized geometries. Given that our parametrization is specifically designed for OPV-related applications, where optimized geometries and stacking patterns are of primary importance rather than broad general-purpose applicability, we opted for a parametrization of sulfur that ensures reliable structural predictions in OPV systems.

In addition, the determination of the free atom eigenenergies ϵ_μ_^free^ of unoccupied orbitals, specifically the 3*d*-orbitals of S and Cl, was based on Bayesian optimization. To this end, the *Bayesian Optimization* package^54^ was used to implement the optimization process. The density of states (DOS) around the Fermi levels for a representative set of the OPV molecules (which will be described later in Section 3.1) was selected as the loss function for tuning the on-site energies. The method for sampling the DOS is detailed in our previous work.^55^ After optimizing the on-site energies of the 3*d*-shells of S and Cl, DFTB DOS of the OPV molecules have good agreements compared with the corresponding DFT results (as shown in Figure S6).

#### 2.2.2 Repulsive Parameters

The repulsive potential is represented as functions of diatomic distances for various element pairs. In practice, this potential is fitted using a combination of exponential, spline, and damping functions, as described in eq 11.

11 $$V_{\mathrm{AB}}\left(R_{\mathrm{AB}}\right)=\{\begin{array}{l} e^{-a_{1}R_{\mathrm{AB}}+a_{2}}+a_{3},,R_{\mathrm{AB}}<R_{\mathrm{AB},0}\\ \sum_{i=0}^{3}b_{i,n}{\left(R_{\mathrm{AB}}-R_{\mathrm{AB},n}\right)}^{i},,R_{\mathrm{AB},n}\leq R_{\mathrm{AB}}<R_{\mathrm{AB},n+1}\\ \sum_{i=0}^{5}c_{i}{\left(R_{\mathrm{AB}}-R_{\mathrm{AB},d}\right)}^{i},,R_{\mathrm{AB},d}\leq R_{\mathrm{AB}}<R_{\mathrm{AB},\mathrm{cutoff}}\\ 0,,R_{\mathrm{AB},\mathrm{cutoff}}\leq R_{\mathrm{AB}} \end{array}$$

where *R*_*AB*,0_, *R*_*AB*,d_, and *R*_*AB*,cutoff_ represent the cutoff of the exponential function, the start of the damping tail, and the cutoff of the repulsive potential, respectively; and *R*_*AB*,*n*_ is the knot of interval *n*. For short distances, typically less than the bonding region, the repulsive potential is described by exponential functions. For the bonding region and longer distances, spline functions are employed to determine the repulsive parameters. Close to the cutoff distance, the repulsive potential is smoothly damped to zero by a fifth-order polynomial. Reference calculations were performed on small molecules with various bond lengths for different chemical bonds using DFT with the corresponding functionals. Using the repulsive fitting code from *SkProgs*,^49^ the repulsive parameters were then generated by fitting the total energies from DFTB to those obtained from DFT, based on this reference data set. The specific molecules used in the reference data set for each element are listed in Table 2.

**Table 2:** Small Molecules Used for Repulsive Fitting

elements	molecules
H	H_2_
C	CH_4_, C_2_H_6_
N	NH_3_, N_2_, CH_5_N
O	H_2_O, O_2_, CO_2_, N_2_O
F	HF, F_2_, CH_3_F, NH_2_F, OF_2_
S	SH_2_, S_2_, CS_2_, N_2_S, SO_2_, SHF
Cl	HCl, Cl_2_, CH_3_Cl, NH_2_Cl, ClOH, ClF, ClSH

### 2.3 Computational Details

#### 2.3.1 DFT and DFTB Calculations for Repulsive Parametrization

The DFT reference calculations were carried out using the *NWChem* code in version 7.2.0.^56^ For the DFT calculations, we used the same range-separation parameters as during the DFTB parametrization. The DFTB calculations employed the development version of *DFTB+*, as can be found in the main branch of the official repository.^57^ Default parameters were used whenever not stated.

#### 2.3.2 DFT and DFTB Calculations for OPV Applications

In DFT calculations, geometry optimizations of single molecules without side chains employed the B3LYP functional along with the 6–31G**basis set and the DFT-D3BJ dispersion model. For single-point DFT and TD-DFT calculations, three different functionals (B3LYP, CAM-B3LYP, and LC-BNL) were applied, using the range-separation parameters as described in Section 2.2.1 in conjunction with the 6–311G**basis set.

In DFTB calculations, geometry optimizations were performed using a rational function-based optimizer with a lower limit of the diagonal Hessian elements set to 0.01 Ha/a_0_^2^. For single molecules, these optimizations were conducted without a dispersion model. For dimer systems, the D3 dispersion model,^58,59^ parametrized according to a previous study,^40^ was employed. It is important to note that using the D3 parameters originally determined for an LC-BNL DFTB parametrization is strictly speaking not fully consistent, as the optimal D3 dispersion parameters might slightly differ between various DFTB parametrizations. At a later stage of our work, we have refitted the D3 parameters for our newly developed DFTB parameter sets using the S66, S66a8, and S66x8 benchmark data sets^60,61^ (see Table S2). Our calculations indicate that the geometry of the BTR-Cl/Y6 dimer does not change significantly between the two D3-parametrizations, and consequently also, the resulting absorption spectra are almost identical (see Figure S7), demonstrating that both parametrizations yield the same OPV-physics. Below we report our original results using the D3-parameters from ref 40., but for completeness we give these refitted D3 parameters in Table S2, which should be used for full consistency in all future calculations with the new parameter sets.

For excited-state property calculations, the linear-response TD-DFTB^62^ and real-time TD-DFTB^63^ formalisms were used. The linear-response TD-DFTB calculations were implemented using the Casida calculator with a Stratmann diagonalizer, and a tailored version of *DFTB+* was used.^64^ Besides, absorption spectra calculations within real-time TD-DFTB were performed using 20,000 steps of electron dynamics with a time step of 0.2 au, applying a Dirac-delta perturbation in three Cartesian directions under an electric field strength of 0.001 V/Å. For electron dynamics driven by external fields, 160000 steps were performed with a time step of 0.02 au, using a continuously pulsed laser aligned with the maximum polarization direction. The electric field strength was set to 0.001 V/Å. Moreover, calculations accounting for solvent effects employed a generalized Born (GB) implicit solvation model,^65^ with specific parameters provided.^66^ In addition, molecular orbitals were visualized by *VMD*,^67^ using values of 0.006 au and 0.005 au to create iso-surfaces for DFT and DFTB results, respectively. Molecular structures shown in the Supporting Information (Figure S1) were visualized by *Avogadro*.^68^ To classify an excited state as either a local excitation or a CT excitation, the charge transfer numbers^69,70^ were computed using the *TheoDORE* package.^71^ During these calculations, the dimer system was partitioned into two fragments, corresponding to the donor and acceptor molecules, respectively.

In xTB-related calculations, geometry optimizations and energy level evaluations were performed using the GFN2-xTB model^72^ within the *DFTB+* program, employing the *tblite* library.^73^ The rational function-based optimizer was used, with a lower limit of the diagonal Hessian elements set to 0.01 Ha/a_0_^2^. Additionally, absorption spectra were computed using the sTDA-xTB method,^74^ employing *xtb4stda* in conjunction with the *stda* program (version *v1.6.3*).^75^

## 3 Results and Discussion

In this section, the proposed parametrization is evaluated and applied to specific targets, i.e., the organic molecules used in photovoltaic active layers. First, both of the electronic and repulsive parameters were assessed using a set of OPV donor and acceptor molecules. For the electronic parameters, properties such as the HOMO–LUMO gap and molecular orbitals were compared with DFT results. The repulsive parameters were tested by comparing bond lengths and vibrational frequencies of optimized geometries from DFT and DFTB. Second, both ground-state and excited-state properties of dimer systems consisting of more than 400 atoms were studied using DFTB with the proposed parametrizations.

### 3.1 Parametrization Scheme

The parametrization scheme for hybrid DFTB is summarized in Figure 1. A previous study^38^ introduced the *ob2–1–1* parametrization using the LC-BNL functional, which included parameters for the elements H, C, N, and O. In this work, the additional elements F, S, and Cl, which are commonly present in OPV molecules, have been parametrized. Moreover, two different functionals B3LYP, and CAM-B3LYP′ (using nonstandard range-separation parameters) were employed. Further details concerning the parametrization process were discussed in Section 2.2.

**Figure 1:**
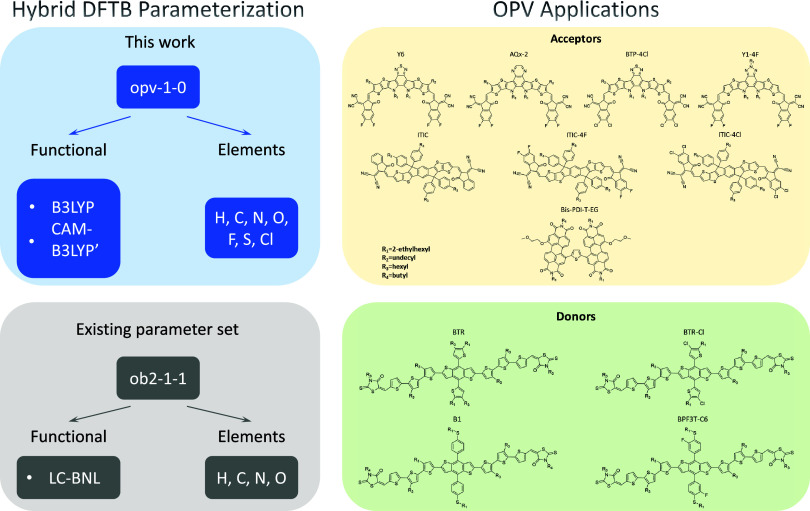
Parametrization scheme for hybrid DFTB and its application to organic photovoltaic (OPV) molecules. The comparison of an existing parameter set and this work is shown in the left panel. The chemical structures of OPV donors and acceptors tested in this work are listed in the right panels.

Additionally, a small data set consisting of small-molecule donors and nonfullerene acceptors^76^ was created to evaluate the new parameters (as shown in the right panels of Figure 1). The data set includes eight acceptors, such as Y6 and its derivatives,^8,77–79^ ITIC and its derivatives,^44,80,81^ and a PDI-based molecule,^82^ as well as four small-molecule donors: BTR,^45^ BTR-Cl,^46^ B1,^83^ and BPF3T-C6.^84^ Single molecules without side-chains were used to evaluate the new parametrization by benchmarking against DFT results, while the complete molecules with side-chains were used to construct dimer systems for realistic applications. Moreover, the experimental HOMO/LUMO-level positions were also collected from the literature, where they were measured using the cyclic voltammetry technique. The resulting gap values can be considered as an approximation of the fundamental gap. It is important to note that those experimental gap values are used exclusively for comparison and to assess the accuracy of different computational methods, since we fit using the corresponding DFT values as references.

### 3.2 Evaluation of Parametrization

#### 3.2.1 Ground State Properties

In this subsection, the HOMO–LUMO gaps and the characteristics of molecular orbitals are selected as ground-state properties for evaluation. DFT-optimized and DFTB-optimized geometries were employed for DFT and DFTB calculations, respectively, to make an overall comparison. Figures 2 and 3 present a comparison of HOMO–LUMO gaps for acceptors and donors using the B3LYP and CAM-B3LYP′ parametrizations, respectively. Results obtained by LC-BNL are shown in Supporting Information, Figures S8 and S9 for comparison purposes. When comparing DFT and DFTB results, the trend in HOMO–LUMO gaps across different acceptors and donors from DFTB calculations aligns well with the DFT references, although there are quantitative differences in the absolute values. For example, in the case of the Y6 family displayed in Figure 2, DFT calculations with both functionals consistently show that AQx-2 has the largest HOMO–LUMO gap, followed by Y6 and BTP-4Cl, with Y1–4F exhibiting the smallest gap. This same trend is observed in the corresponding DFTB calculations. For the ITIC family of acceptors and the four BDT-core based donors, both DFT and DFTB calculations reveal consistent trends in the HOMO–LUMO gaps, following the order: ITIC > ITIC-4F > ITIC-4Cl for the acceptors (Figure 2), and BPF3T-C6 > BTR-Cl > B1 > BTR for the donors (Figure 3). Besides, the downshifting trend of energy levels resulting from the introduction of F or Cl atoms, as observed in the DFTB calculations, is consistent with experimental results. For example, this trend is evident in the transition from the molecule ITIC to ITIC-4F and ITIC-4Cl. Moreover, the relative differences in HOMO–LUMO gaps between different molecules in DFTB results are close to those from DFT. For example, the difference in HOMO–LUMO gaps between ITIC and ITIC-Cl is 0.05 eV according to DFT calculations using both two functionals. The corresponding difference is 0.09 eV when using B3LYP DFTB, and 0.07 eV when using CAM-B3LYP′ DFTB. When comparing experimental results with calculations, most acceptor molecules show a good agreement between experimental trends and computational predictions. However, the agreement is not as strong for donor molecules. This disagreement may be attributed to the measurement methods used in the experiments and the solvent effects, which can influence the geometry and packing of molecules in solution, differing from calculations performed on isolated molecules. Moreover, when comparing parametrizations using different functionals, it is evident that the values calculated using the B3LYP functional most closely align with the experimental results. As the fraction of HF exchange increases, progressing from B3LYP to CAM-B3LYP′ and then to LC-BNL (as shown in Figures S8 and S9), the HOMO–LUMO gaps are continuously opened. The overall trend across the different functionals, however, is consistent.

**Figure 2:**
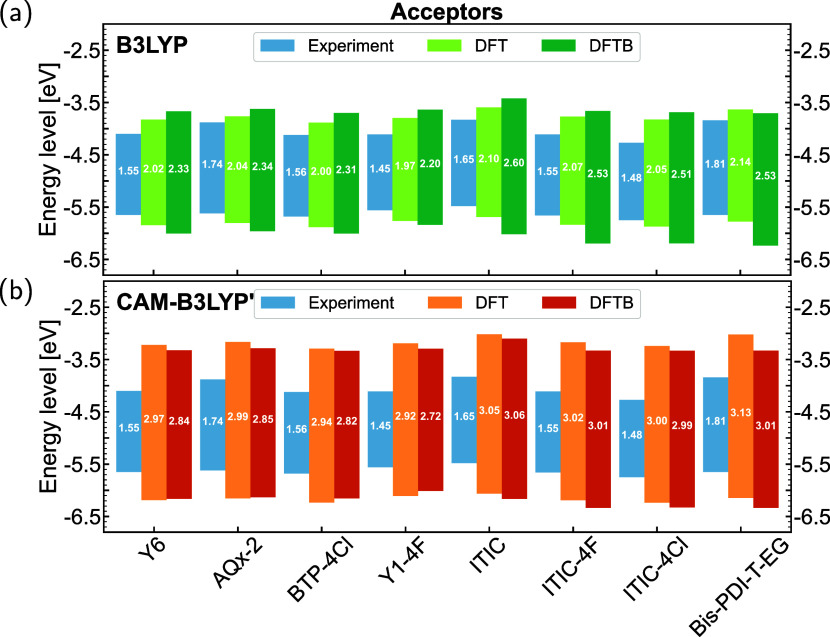
HOMO/LUMO-level positions and gap sizes (in eV) of OPV acceptor molecules calculated by DFT and DFTB using (a) B3LYP, and (b) CAM-B3LYP′.

**Figure 3:**
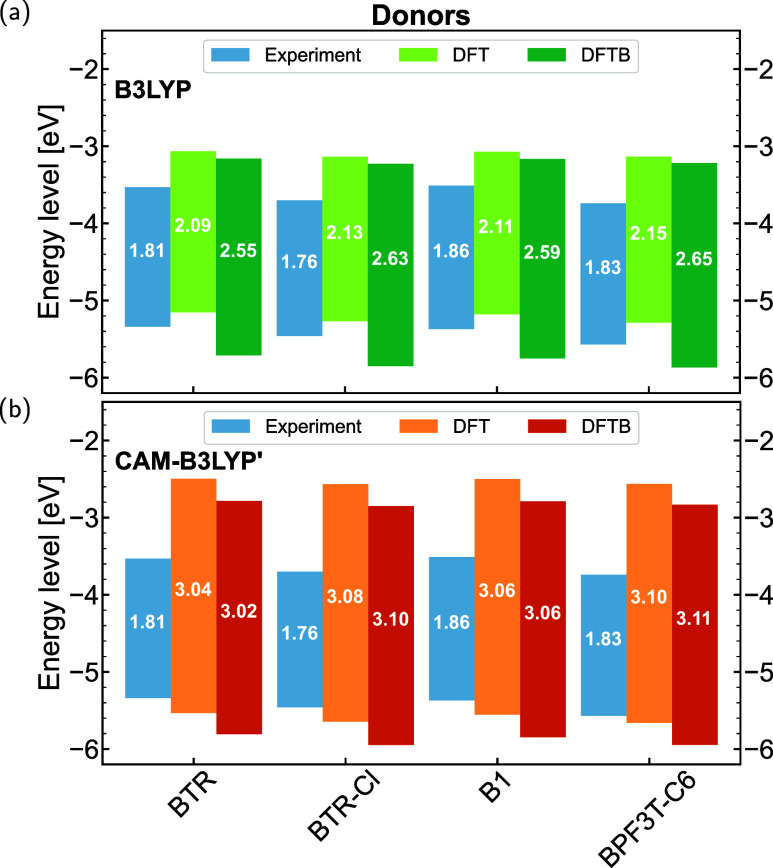
HOMO/LUMO-level positions and gap sizes (in eV) of OPV donor molecules calculated by DFT and DFTB using (a) B3LYP, and (b) CAM-B3LYP′.

Another ground state property evaluated as part of this study is the frontier molecular orbital. The comparisons of orbitals obtained by DFTB and DFT approaches are presented in Figures 4, S10, and S11. For the acceptor molecule Y6 (Figure 4(a)), DFT calculations show that the HOMO orbital is well localized at the central core, which consists of two symmetrical electron-donating (D) units and one electron-deficient (A′) unit, with less population in the electron-withdrawing (A) ending groups. On the other hand, the LUMO orbital is delocalized over the entire backbone. For the donor molecule BTR-Cl (Figure 4(b)), the HOMO and the LUMO orbitals are well delocalized along the main chain of building blocks. These key features observed in DFT calculations are correctly reproduced by DFTB.

**Figure 4:**
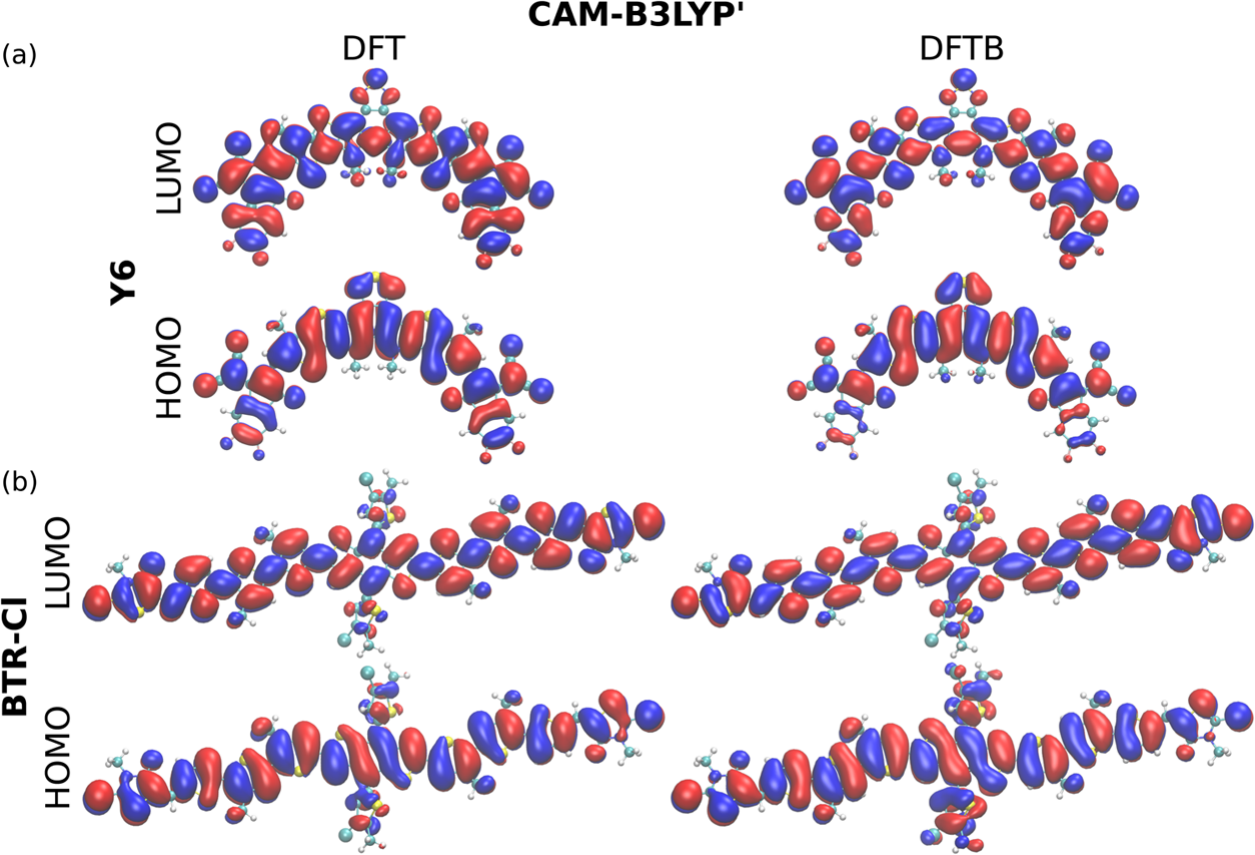
Visualization of the HOMO and LUMO orbitals of (a) the acceptor molecule Y6; and (b) the donor molecule BTR-Cl, as computed on the CAM-B3LYP′-DFT and -DFTB levels of theory. Blue and red colors indicate positive and negative iso-surfaces. Orbitals are visualized by *VMD*,^67^ using values of 0.006 au and 0.005 au to create iso-surfaces for DFT and DFTB results, respectively.

#### 3.2.2 Repulsive Evaluation

To evaluate the repulsive parameters, relaxed geometries of molecules were obtained from DFT and DFTB calculations. Bond lengths were first selected as a target for assessing the quality of the DFTB geometries and the effectiveness of the repulsive parameters. As the chemical environments and bond types in the small molecules used for repulsive potential fitting (Table 2) are different from those typically found in OPV systems, it is essential to assess carefully the reliability and transferability of the fitted repulsive parameters. However, it should be noted that the repulsive potential does not explicitly depend (in leading order) on the bond types, but only on the reference densities of the interacting atoms. We have constructed a supplementary data set comprising small molecules that more closely resemble the chemical environments present in OPV materials. We then analyzed the bond lengths of various chemical bonds in this data set using different methods and compared the results with DFT references. As shown in Table S3, bonds that were not included in the original fitting set, such as those involving sp^2^-hybridized carbon atoms bonded to other elements, are well reproduced by our parametrizations, demonstrating good agreement with DFT reference values. Furthermore, key quantitative trends among different bond types are captured accurately. For example, the bond length of the sp^2^S–sp^2^C bond in thiophene (C_4_H_4_S) is shorter than that of the sp^3^S–sp^2^C bond in benzenethiol (C_6_H_5_SH), in agreement with chemical intuition and DFT results. Similarly, the shorter bond length of the sp^2^C–sp^2^C bond in ethylene (C_2_H_4_) compared to the sp^3^C–sp^3^C bond in ethane (C_2_H_6_) is correctly reproduced.

Subsequently, we applied our repulsive parameters to the OPV-specific data set to further evaluate their transferability to the target systems of interest. Several important chemical bonds present in OPV molecules were targeted, and the bond lengths in different molecules were collected for comparison. Results from the CAM-B3LYP′ DFTB parametrization are presented below, while those obtained using the B3LYP parametrization are provided in the Supporting Information (Table S4 and Figure S12). Both parametrizations demonstrate comparable accuracy. Table 3 presents the statistical analysis of bond lengths, showing the average length and standard deviation of the selected bonds across various molecules. The results indicate that our CAM-B3LYP′ parametrization tends to slightly overestimate the lengths of C–C, C=S, and C–F bonds, with the largest absolute error being 0.03 Å in the average bond length. Conversely, it underestimates the C≡N bond length by 0.03 Å on average. In addition, the standard deviations of bond lengths obtained from DFTB calculations have similar values as DFT results, indicating that the influence of the chemical environment on bond lengths exhibits a consistent trend for both methods. The largest absolute deviation among different chemical bonds is 0.046 Å, observed in the case of the C–C bond. Overall, the optimized geometries of CAM-B3LYP′ DFTB still exhibit reasonable bond lengths, confirming the reliability of the repulsive parameters.

**Table 3:** Comparison of the Average Bond Length (MEAN) and Its Standard Deviation (STD) of OPV Molecules in the Dataset, as Measured for Geometries Relaxed Using B3LYP DFT and the CAM-B3LYP′ DFTB Parametrization of this Work^a^

bond	MEAN (DFT)	STD (DFT)	MEAN (DFTB)	STD (DFTB)	MAD	MAX	10th	90th
C–C	1.46	0.041	1.49	0.040	0.030	0.046	0.021	0.039
C=C	1.38	0.006	1.38	0.005	0.003	0.005	0.000	0.005
C≡N	1.17	0.000	1.14	0.001	0.030	0.030	0.029	0.030
C=O	1.22	0.001	1.23	0.005	0.008	0.012	0.003	0.012
C=S	1.65	0.000	1.68	0.000	0.027	0.027	0.027	0.027
C–F	1.34	0.005	1.37	0.003	0.028	0.030	0.026	0.030
C–Cl	1.74	0.004	1.74	0.003	0.003	0.004	0.002	0.003

a Mean absolute deviation (MAD), maximum absolute deviation (MAX), 10th and 90th percentiles of the deviation between DFT and DFTB calculated bond lengths are listed. All quantities are provided in angstrom (Å).

Solving the harmonic vibrational problem provides another stringent test of the repulsive parameters. Figure 5 shows a comparison of vibrational frequencies between CAM-B3LYP′ DFTB and B3LYP DFT reference calculations for two example molecules. The DFTB results align closely with the DFT reference, exhibiting vibrational modes with similar frequencies. For example, the highest frequency calculated for BTR-Cl by DFT and DFTB is 3269 and 3298 cm^–1^, respectively, with an absolute error of 29 cm^–1^. Similarly, the highest frequency for Y6 shows an absolute error of 43 cm^–1^. Furthermore, the vibrational frequencies derived from the DFTB Hessian matrix (Figure 5) can be correlated with the frequencies obtained from the velocity-velocity autocorrelation functions computed during molecular dynamics simulations (Figure S13). In summary, the repulsive parameters have been evaluated from two perspectives, and the presented parametrization is able to produce reasonably optimized geometries for OPV molecules.

**Figure 5:**
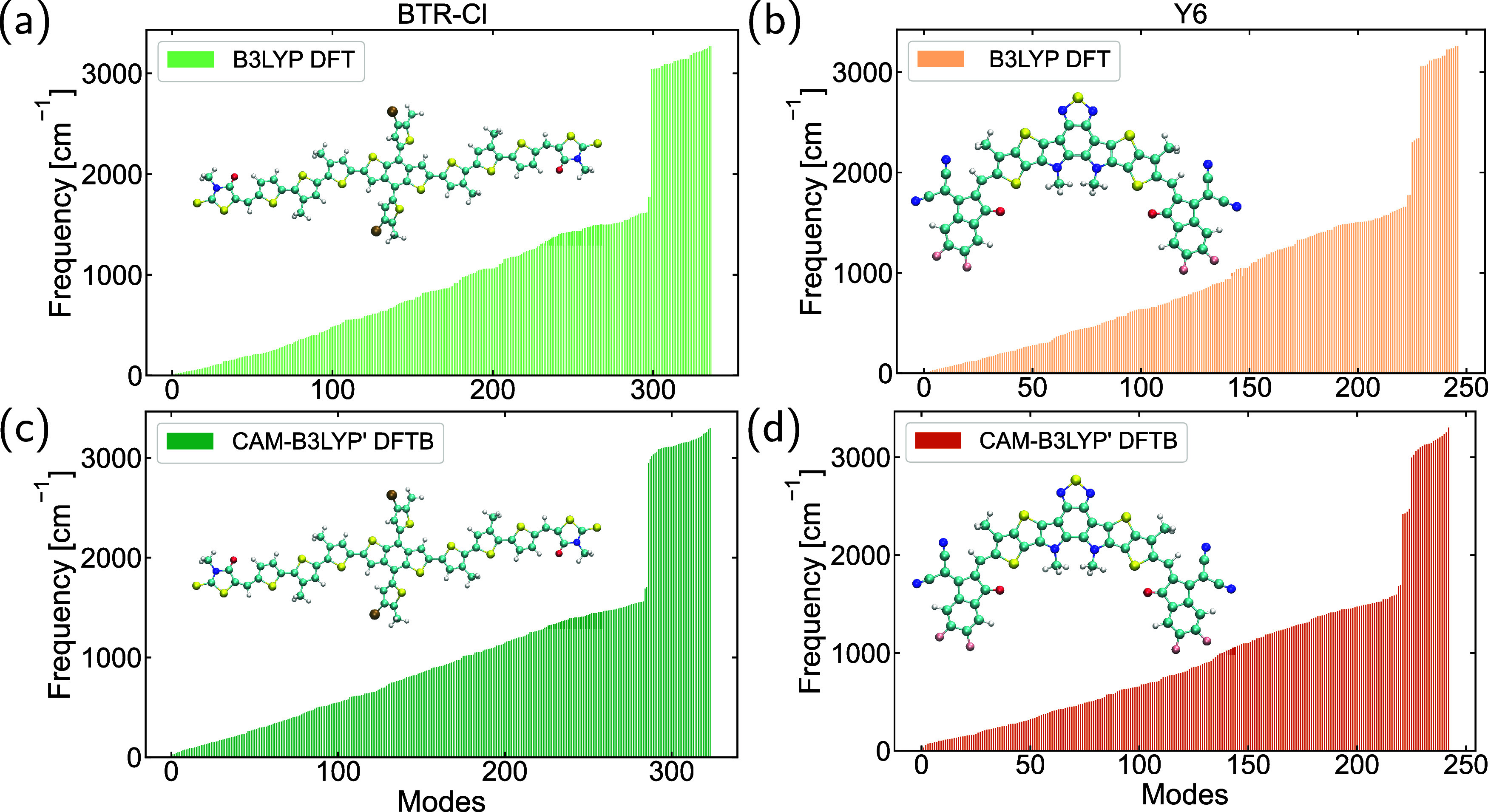
Comparison of the vibrational modes of the donor molecule BTR-Cl, based on (a) B3LYP DFT and (c) CAM-B3LYP′ DFTB; and the acceptor molecule Y6 based on (b) B3LYP DFT and (d) CAM-B3LYP′ DFTB (relaxed geometries shown in the inset).

#### 3.2.3 Bench-Marking against Other Approximate DFT Methods

It is insightful to compare the performance of currently available approximate DFT methods for OPV applications to identify potential limitations. Here we selected two representative properties for comparison: the HOMO/LUMO energy level positions and gap sizes for ground-state characteristics, and absorption spectra for excited-state properties. We compared the performance of our new DFTB parametrizations against DFTB3 with the *3ob-3–1* parameter set^29,85,86^ and GFN2-xTB.^72^ Both DFTB3 and GFN2-xTB provide parameters for the elements present in the OPV molecules. However, it is important to note that neither method incorporates long-range corrections, which is expected to limit their accuracy in OPV-related calculations. Our comparison with these two methods is primarily due to the availability of their parameters rather than their underlying theoretical framework.

Results related to the HOMO–LUMO gaps are presented in Figures S14–S17. As previously discussed, DFTB with the newly developed parametrization exhibits consistency with its DFT reference, particularly in capturing trends within molecular families. The same set of OPV donor and acceptor molecules was analyzed using the DFTB3 and GFN2-xTB methods, with HOMO–LUMO gaps evaluated based on the optimized geometries obtained from each method, respectively. Figures S14 and S15 reveal that, although the HOMO/LUMO energy levels and gap sizes computed with the *3ob-3–1* parametrization are closer to experimental values compared to those obtained with the B3LYP functional, certain critical features and trends among different molecules are not accurately reproduced. For example, the expected trend within the ITIC family of acceptor molecules (ITIC > ITIC-4F > ITIC-4Cl) is absent in the results from the *3ob-3–1* parametrization. Additionally, discrepancies arise in the relative gap differences between donor and acceptor molecules. For example, the HOMO–LUMO gap of the donor molecule BTR-Cl is experimentally larger than that of the acceptor molecule Y6, a trend correctly captured by both DFT and DFTB using the B3LYP functional. However, the *3ob-3–1* parametrization incorrectly predicts the opposite relationship. This deviation in DFTB3 results with the *3ob-3–1* parametrization limits its reliability for rapid molecular screening.

For the GFN2-xTB methods, Figures S16 and S17 indicate that the HOMO/LUMO energy levels are not well-aligned with either experimental or DFT­(B) results, exhibiting substantial shifts in absolute energy values. Moreover, the expected trends among different molecules are not captured using the GFN2-xTB method. These findings suggest that GFN2-xTB is not well-suited for such electronic property evaluations, as the method is primarily designed for structural optimizations and noncovalent interaction energy calculations.

Furthermore, we examined and benchmarked absorption spectra. As shown in Figure S18, the *3ob-3–1* parametrization leads to red shifts compared to the CAM-B3LYP′ DFT reference. Notably, for the donor molecule BTR-Cl, the *3ob-3–1* parametrization predicts absorption peaks above 800 nm, which are nonphysical. In contrast, the sTDA-xTB method exhibits blue shifts in its predictions (Figure S19). The absence of long-range correction in both methods likely contributes to their poor performance in predicting excited-state properties.

### 3.3 Application on All-Small-Molecule OPV Dimer Systems

Following the validation of the proposed parameters through calculations on OPV molecules, an investigation was conducted on donor–acceptor dimer systems. The small-molecule donor BTR-Cl and the nonfullerene acceptor Y6 were selected to construct the dimer system. At first, ground and excited state properties were analyzed to ensure the compatibility of the donor and acceptor molecules. Subsequently, the dimer system was studied using real-time TD-DFTB to demonstrate the CT excitation within the OPV system.

#### 3.3.1 Compatibility between Donor and Acceptor

In order to realize a highly efficient OPV system, the compatibility between the donor and acceptor material is crucial. Here, compatibility was checked for two aspects: energy levels and absorption characteristics. The energy level alignment directly influences the driving force for exciton dissociation and CT, both of which are critical determinants of the overall PCE of the OPV device.^87^ As noted in another study,^88^ an ionization energy (IE) offset of approximately 0.5 eV between the donor and nonfullerene acceptor can enhance exciton-to-charge conversion efficiency in OPV systems. Fluorinated nonfullerene acceptors, such as Y6, typically exhibit deeper HOMO and LUMO levels due to the introduction of electronegative atoms like F, compared to nonfluorinated ones. Therefore, it is essential that the energy level of the donor molecule is appropriately matched. Figure 6(a) illustrates the energy levels of the donor–acceptor pair (BTR-Cl and Y6), as determined by various methods. DFTB calculations indicate a ΔHOMO value of 0.5 eV, which, although slightly underestimated relative to the DFT calculations, aligns more closely with experimental results. Furthermore, the theoretically calculated ΔHOMO value supports the high PCE observed in experiments.

**Figure 6:**
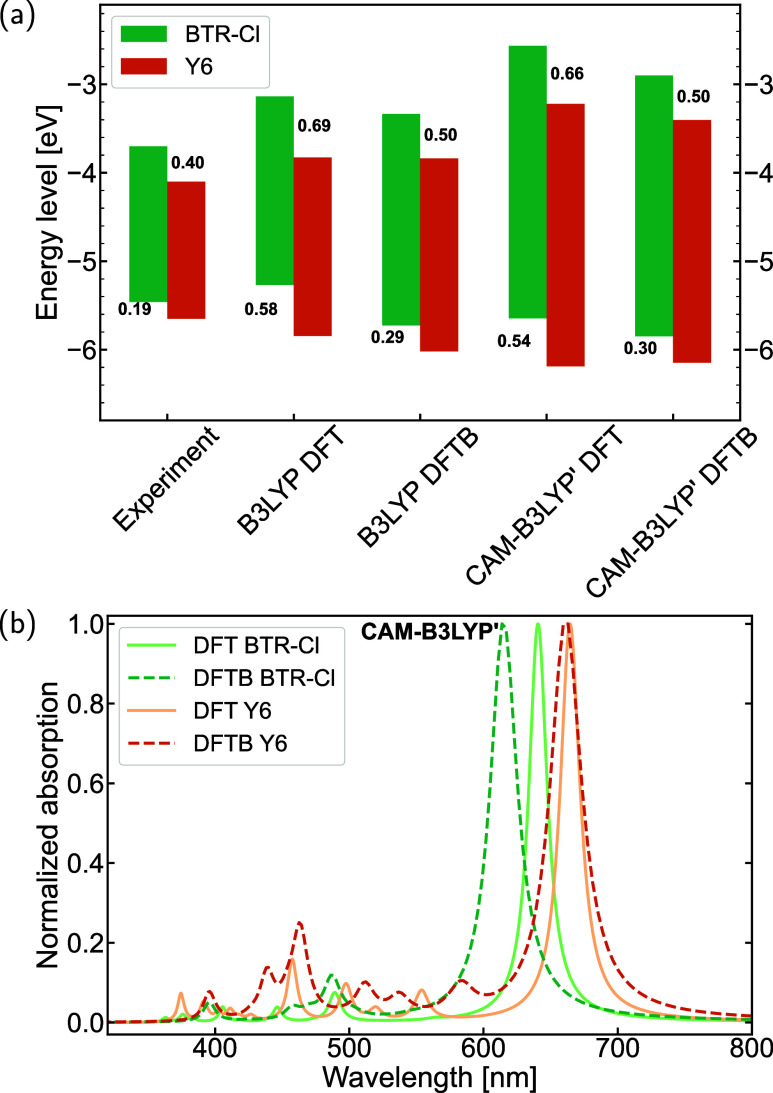
(a) HOMO/LUMO-level positions of a donor–acceptor pair (BTR-Cl and Y6) obtained by different methods, with the numbers at the bars indicating the relative LUMO–LUMO and HOMO–HOMO energy difference (eV) between the donor and the acceptor molecule; (b) comparison of normalized absorption spectra of BTR-Cl and Y6 single molecule calculated by CAM-B3LYP′ linear response TD-DFT and TD-DFTB.

Another aspect of compatibility was examined through the absorption spectra of both molecules, as shown in Figure 6(b). Note that these calculations were performed on single molecules without side-chains, and the effects of the solvent were not considered. The agreement between DFT and DFTB results for the acceptor Y6 is better than for the donor molecule. For BTR-Cl, the DFTB calculation shows a blue shift of 27 nm (∼0.09 eV) in the absorption peak, though it remains within a reasonable range. To further refine the analysis, DFTB calculations were extended to cover the complete molecule with side-chains, and an implicit solvation model for chloroform was applied, making the results more comparable to experimental data. As shown in Figure S20, the solvent had only a minimal effect on BTR-Cl, causing a red shift of 6 nm (∼0.02 eV) after applying the implicit solvation model. In contrast, the solvent effect causes a red shift of 44 nm (∼0.12 eV) in the absorption peak for Y6, which is closer to the experimental results. Moreover, the difference in the absorption peak wavelengths between the donor and acceptor molecules can enhance the light absorption efficiency of the OPV device. Overall, the compatibility between the donor and acceptor materials was investigated using different methods. The comparative analysis indicates that DFTB with the new parametrization is an effective tool for assessing the compatibility of novel donor–acceptor pairs.

#### 3.3.2 Investigation of the Dimer System by TD-DFTB

The initial dimer structure, consisting of 435 atoms, was constructed by stacking the DFTB-optimized geometries of the donor and acceptor molecules (including side-chains) in a face-on configuration, with an initially large intermolecular distance (approximately 10.0 Å) and favorable π–π overlap. Subsequently, geometry optimization was performed using DFTB with the CAM-B3LYP′ parameter set and the D3 dispersion correction model^58,59^ (as parametrized in a previous study^40^), yielding the final dimer structure employed in our simulations. The optimized geometry of the dimer system is provided in the Supporting Information. First, we compared the absorption spectra computed using different methods and parametrizations, employing the same CAM-B3LYP′ DFTB optimized geometry for consistency. The absorption spectra of the isolated donor molecule BTR-Cl, the acceptor molecule Y6 (both with side-chains), and the corresponding dimer system were compared and analyzed. As shown in Figure 7(a), the CAM-B3LYP′ DFTB calculations reveal that the main absorption peak position of the donor remains unchanged, while that of the acceptor undergoes a subtle red shift. Notably, a new peak appears at 799.6 nm, which is absent in the spectra of the isolated donor and acceptor. This phenomenon is likely related to a CT excitation occurring in the donor–acceptor system. Additional spectra obtained using other methods are presented in Figure S21. The *3ob-3–1* parametrization^29,85,86^ employing the PBE functional without long-range corrections predicts an absorption peak for the donor material above 800 nm, which is an unphysical outcome (Figure S21­(a)). Furthermore, it fails to capture any CT-related excitation. Similarly, the performance of the sTDA-xTB method was evaluated (Figure S22), and no evidence of CT-related excitation was observed as well. In contrast, the three new parametrizations that incorporate hybrid functionals provide good predictions of the absorption spectra for the target system, with the CT-related excitation observable in the spectra. However, a notable difference is observed in the CT-related excitation wavelengths: the B3LYP parametrization predicted a blue-shifted peak at 737.2 nm compared to CAM-B3LYP′ (799.6 nm) and LC-BNL (787.0 nm). Moreover, the absorption spectrum computed using the B3LYP parametrization shows a splitting of the main peak into two distinct components, with a secondary peak emerging at 549 nm (Figure S21­(b)). This secondary peak will be studied and analyzed in a later section. Such a phenomenon is not observed with the CAM-B3LYP′ or LC-BNL parametrizations, suggesting potential limitations of the B3LYP-based parameter set. Based on these results, we selected the CAM-B3LYP′ parametrization for subsequent studies.

**Figure 7:**
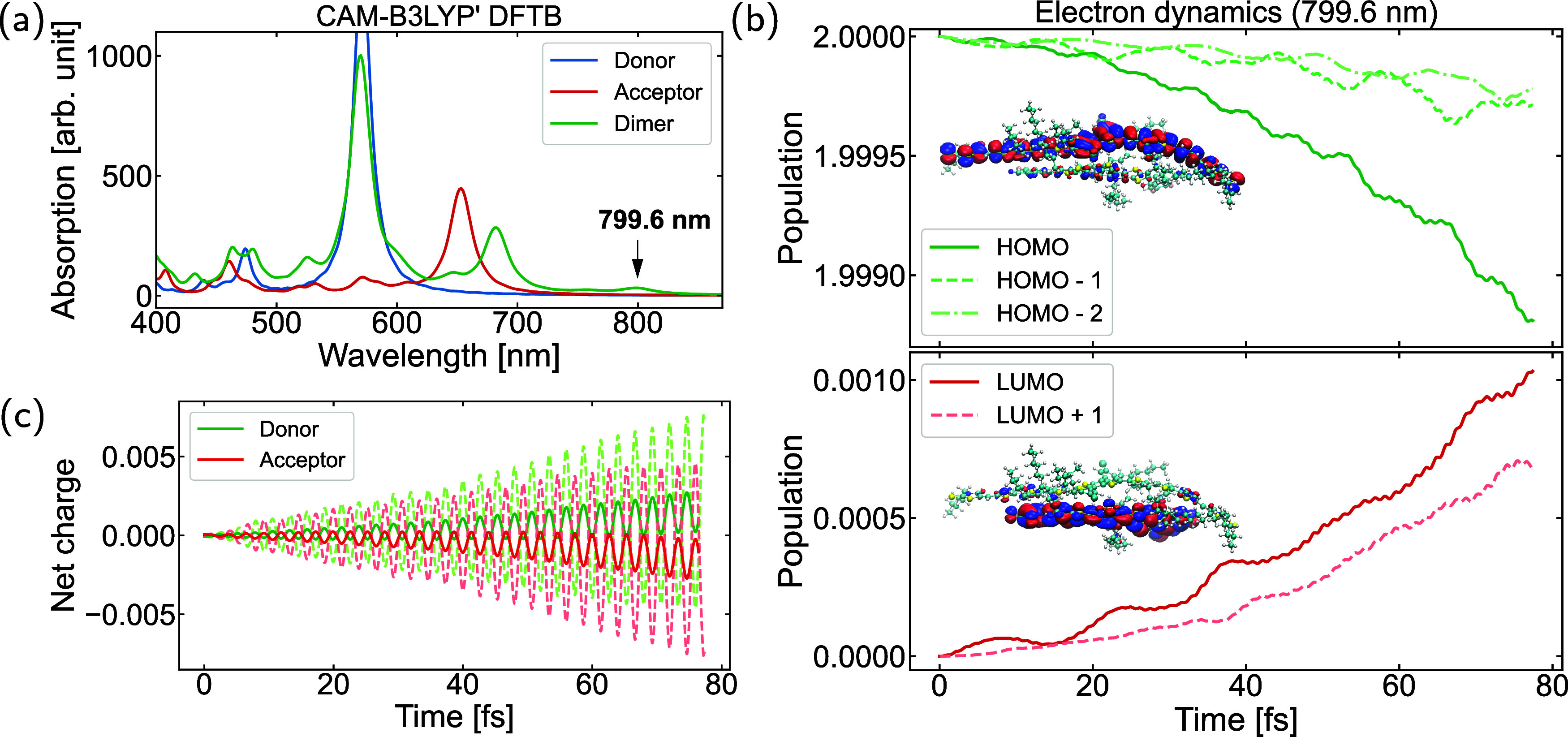
(a) Absorption spectra of the isolated donor molecule BTR-Cl, the acceptor molecule Y6 (both with side-chains) and the corresponding dimer system, from CAM-B3LYP′ real-time TD-DFTB; (b) populations projected onto the ground state molecular orbitals, obtained by electron dynamics using CAM-B3LYP′ DFTB at 799.6 nm; (c) charge-transfer between the donor and acceptor molecules during the electron dynamics simulation. Dashed lines represent the instantaneous net charge variation on each molecule as a function of time, while solid lines denote the corresponding running average.

CT excitation typically refers to an excitation process transferring an electron from one molecular region to another.^89,90^ In our case, the excitation involves an intermolecular CT from the donor molecule to the acceptor molecule. To verify this, electron dynamics driven by an external field were employed. A continuously pulsed laser with an energy of 1.55 eV (corresponding to the absorption peak at 799.6 nm) and a field strength of 0.001 V/Å was applied along the maximum polarization direction to induce the excitation. Figure 7(b) shows the populations projected onto the ground-state molecular orbitals. A clear trend is observed: a decay in the HOMO population of the dimer system and an accumulation in the LUMO population, indicating a direct CT from the HOMO to the LUMO of the dimer. The insets show the nature of the HOMO and LUMO orbitals, which are localized on the donor and acceptor molecules, respectively. This transition from the HOMO to the LUMO of the dimer is further confirmed by a linear-response TD-DFTB calculation, as presented in Table S6. Furthermore, the charge transfer number computed using the *TheoDORE* package provides clear evidence of a CT excitation (Table S6). A charge transfer number of 0.954 indicates that this transition corresponds to a charge resonance state. Therefore, a direct CT from the donor to the acceptor is confirmed, proving that the peak at 799.6 nm is indeed associated with a CT excitation. Besides, Figure 7(c) displays the net charge changes of both molecules as a function of time, with dashed lines showing the actual values and solid lines representing the running average. This indicates a CT between the donor and acceptor molecules.

In addition, the influence of side-chains on CT excitations was investigated. The same BTR-Cl/Y6 dimer system, using a relaxed geometry (relaxing with side-chains), was examined with all side-chains removed, and it was compared to the system with side-chains intact. Figure 8(a) shows a decrease of approximately 20% in the light absorption intensity of peaks around 800 nm after side-chain removal (between green and red lines), which translates into a weakened CT excitation. To ensure that the specific positions of side-chains did not affect this result, a DFTB molecular dynamics simulation was performed, where the molecular cores were frozen, allowing only the atoms in the side-chains to move. Snapshots representing the geometries with the four lowest total energies were selected for analysis. All four systems exhibited higher light absorption intensity in the CT excitation peak than the one without side-chains, reinforcing the observation that side-chain removal reduces CT excitation. Additionally, Figure 8(b) indicates that the transfer of the HOMO population to the LUMO population in the dimer system exhibits a decreasing trend after removing the side-chains. To further confirm the influence of side-chains, another dimer system (BTR/Y6) was studied using the relaxed geometries with and without side-chains, and a similar reduction in the intensity of CT-related absorption was observed (as shown in Figure S23). These findings suggest that side-chains contribute to the electronic stabilization of CT excitations. This stabilization is particularly relevant for understanding CT mechanisms in dimers and more complex systems, where CT states play a significant role.^38^ In this regard, including the alkyl chains in simulations may enhance the theoretical description of these mechanisms, with the computational efficiency of DFTB offering a suitable framework for such studies. To further investigate the influence and mechanisms of side-chains in photoinduced charge transfer processes, we plan to perform nonadiabatic molecular dynamics simulations in the future, incorporating the effects of nuclear motion. These simulations could be conducted using either the Ehrenfest dynamics or the Surface Hopping method. The former would require extending the current implementation of Ehrenfest dynamics^63^ to incorporate the necessary long-range corrections, while the latter, which has been recently implemented,^91^ demands the execution of a large number of trajectories. Both tasks, however, lie beyond the scope of the present work.

**Figure 8:**
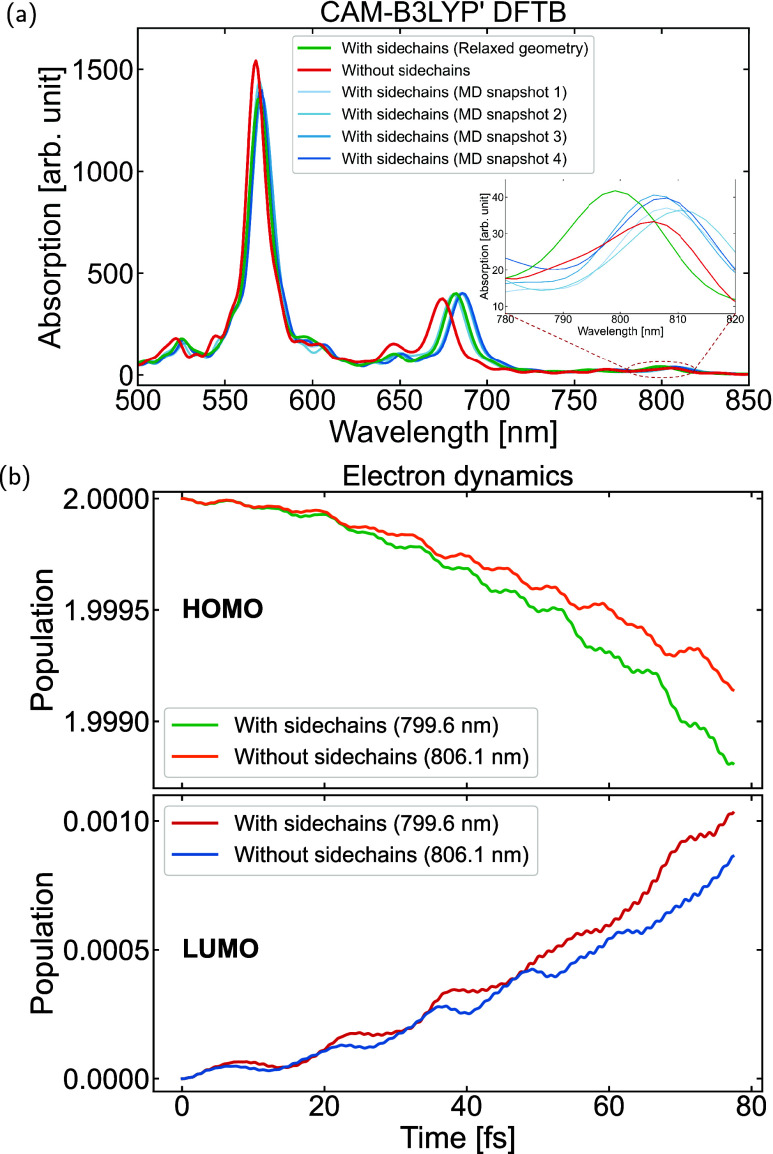
(a) Comparison of the absorption spectra of the BTR-Cl and Y6 dimer system with and without side-chains, using different geometries. The insets display the charge-transfer excitation region on a finer scale; (b) comparison of the populations projected onto the ground state molecular orbitals between relaxed geometry with side-chains and the corresponding system without side-chains during electron dynamics using CAM-B3LYP′ DFTB.

Another critical aspect to explore is the comparison of CT excitation-related results obtained by different functionals within the DFTB framework. In addition to CAM-B3LYP′, we carried out electron dynamics using the B3LYP- and LC-BNL-based parameter sets. As shown in Figures S24 and S25, the intensity of the CT excitation is noticeably weaker when using the B3LYP parametrization, compared to CAM-B3LYP′ and LC-BNL. Moreover, we further investigated the phenomenon of the main absorption peak splitting observed with the B3LYP parametrization, as previously shown in Figure S21­(b). This secondary peak emerging at 549 nm is confirmed by a linear-response TD-DFTB calculation (as detailed in Table S5), which is identified as a transition from orbital 586 to orbital 591. Visualization of the involved orbitals (Figure S26) suggests that this excitation corresponds to a direct CT from the donor to the acceptor. However, the energy associated with this transition is unexpectedly high for such a CT process, raising concerns regarding the physical accuracy of this result. Consequently, CAM-B3LYP′ and LC-BNL parametrizations provide a more reliable description of excited-state properties, as the B3LYP parametrization tends to underestimate CT excitations at lower energies while predicting spurious CT states at higher energies.

## 4 Conclusions

In this work, we present 2 new sets of DFTB parameters tailored for OPV applications. These parameter sets are based on the widely used hybrid functionals B3LYP and CAM-B3LYP′. The included elements: H, C, N, O, F, S, and Cl, cover the essential atoms typically present in the donor and acceptor molecules used in OPV technology. By evaluating a data set of OPV donor and acceptor molecules, we demonstrate that the newly developed parametrizations exhibit consistent performance compared to their respective DFT references. In particular, the B3LYP-based parameter set is recommended for ground-state calculations, as it provides the best performance in predicting HOMO–LUMO gaps compared to experimental results. The accuracy of this set, combined with the efficiency of DFTB, makes it ideally suited for rapid screening of a large set of molecules to assess their compatibility as donor–acceptor pairs. However, the B3LYP-based parameter set tends to underestimate CT excitations, which are crucial for accurately describing excited-state dynamics in these materials. In contrast, the CAM-B3LYP′-based parameter set tends to overestimate the HOMO–LUMO gaps of the studied molecules but provides a more accurate representation of excited states, particularly CT excitations. This makes the CAM-B3LYP′ set the most suitable choice for investigating optical properties and photoinduced CT dynamics using TD-DFTB. Electron dynamics simulations provide clear evidence of CT excitations in donor–acceptor dimers, which produce a CT upon illumination of the system. In addition, our results demonstrate that the molecular side-chains play a significant role in stabilizing these CT excitations.

In summary, the B3LYP-based parameter set is highly effective for ground-state calculations, delivering accurate estimates of HOMO–LUMO gaps and making it well-suited for screening potential donor–acceptor pairs. On the other hand, the CAM-B3LYP′-based set provides a more reliable description of excited-state properties, particularly CT excitations, making it the preferred choice for investigating optical properties and photoinduced CT dynamics. Together, these parametrizations provide complementary capabilities for the computational exploration of materials in OPV using DFTB. In addition, the newly developed parameter sets have the potential transferability to systems exhibiting similar electronic and structural features, particularly those containing conjugated frameworks, making them suitable for applications in chromophores and optoelectronic materials. In the future, we aim to perform nonadiabatic molecular dynamics simulations to incorporate nuclear motion effects into dynamics.

## Supplementary Material





## Notes

Methods: detailed descriptions and discussions related to the parametrization process (Section S1); and supporting results: additional results obtained using the newly developed parameter sets, including HOMO–LUMO gaps; molecular orbitals, repulsive evaluation, and molecular dynamics, it also includes bench-marking against other approximate DFT methods; solvent effect on absorption spectrum; comparison of parametrizations on excited-state properties; influence of side-chains on charge-transfer excitations; and comparison of parametrizations on charge-transfer excitations (Section S2) (PDF)
Data of geometry file and parameter sets (ZIP)
