# Supplementary material for: Hybrid Functional DFTB Parametrizations for Modeling Organic Photovoltaic Systems
Source: J Chem Theory Comput. 2025 May 8;21(10):5103–17. doi: 10.1021/acs.jctc.5c00232 (PMC12120918; doi:10.1021/acs.jctc.5c00232)
Supplement: Supplementary file 1 [file ct5c00232_si_001.pdf]

Supporting Information

Hybrid functional DFTB parametrizations for modeling organic photovoltaic systems

Wenbo Sun,[*,†] Tammo van der Heide,[†] Van-Quan Vuong,[‡] Thomas Frauenheim,[¶,§] Michael A. Sentef,[†] Bálint Aradi,[†] and Carlos R. Lien-Medrano[*,†]

†*Institute for Theoretical Physics and Bremen Center for Computational Materials Science, University of Bremen, 28359 Bremen, Germany*

‡*Institute for Physical Chemistry, Karlsruhe Institute of Technology, 76131 Karlsruhe, Germany*

¶*School of Science, Constructor University, Campus Ring 1, 28759 Bremen, Germany*

§*Institute for Advanced Study, Chengdu University, Chengdu 610106, P. R. China*

E-mail: wsun@uni-bremen.de; cmedrano@uni-bremen.de

This supporting information presents the following contents:

Supporting Sections S1 to S2

Supporting Figures S1 to S26

Supporting Tables S1 to S7

S1 Methods

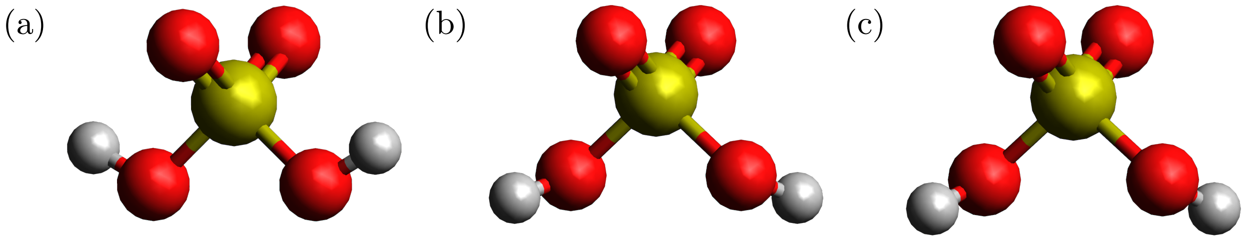

Figure S1: Optimized geometries of a H_2SO_4 molecule obtained by DFTB using (a) a previous LC-BNL parametrization,[1] (b) the *3ob-3-1* set,[2] and (c) the LC-BNL re-parameterization of sulfur in this work, visualized by *Avogadro*.[3]

Table S1: Optimized atomic parameters used to generate electronic parametrization of the LC-BNL functional for different elements. Parameters are given in atomic units ($r^{\mathrm{den}}[\mathrm{a}_0]$, $r^{\mathrm{wf}}[\mathrm{a}_0]$, and $\epsilon_{\mu}^{\mathrm{free}}[\mathrm{Ha}]$).

**Functional**	**Parameters**	**H**	**C**	**N**	**O**	**F**	**S**	**Cl**
LC-BNL	power	2	2	2	2	2	4	9
	r^{den}	3.2	7.6	4.6	5.0	4.0	8.0	9.0
	$r_{\mathrm{sp}}^{\mathrm{wf}}$	3.2	3.8	2.3	2.5	2.8	4.0	3.0
	$r_{\mathrm{d}}^{\mathrm{wf}}(\epsilon_{\mu}^{\mathrm{free}})$						4.5 (0.140)	4.5 (0.062)

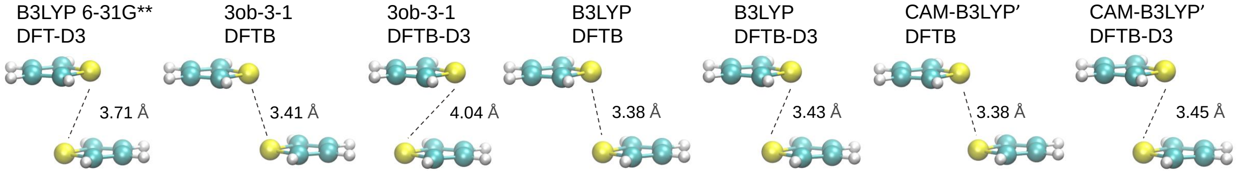

Figure S2: Optimized structures of antiparallel (AP) thiophene dimers at different levels of theory, with the S–S distance provided in Å.

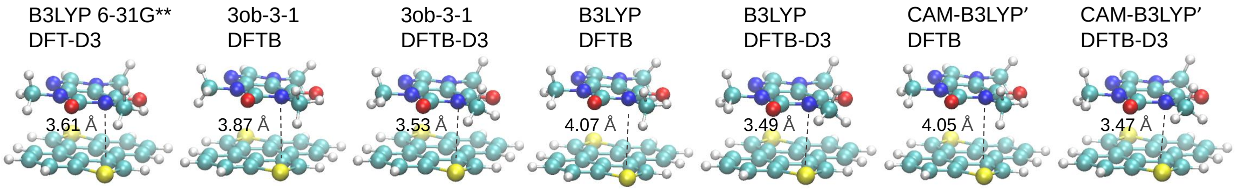

Figure S3: Optimized structures for a representative caffeine sensing system at different levels of theory, with the S–N distance provided in Å.

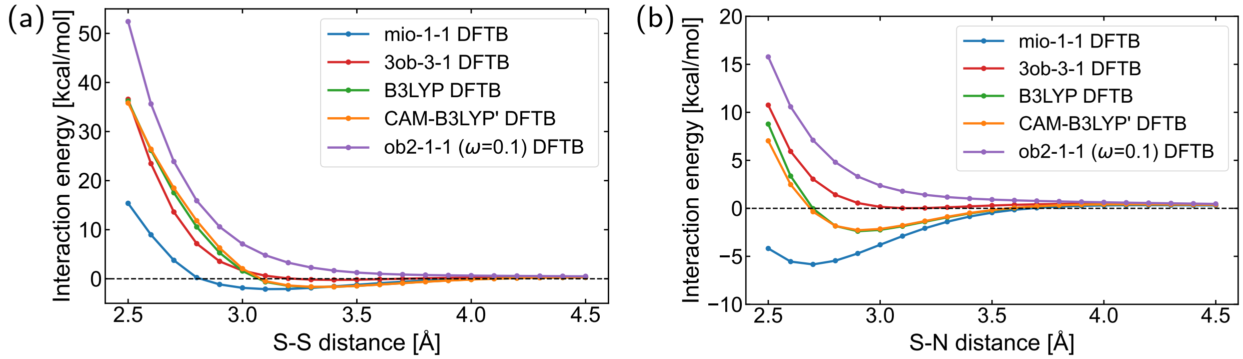

Figure S4: Interaction energy of (a) the H_2S dimer and (b) the H_2S-NH_3 dimer computed using different DFTB parameter sets.

We assessed our parametrization using two example systems from the references:[4,5] the antiparallel (AP) thiophene dimer and a caffeine sensing system.[6] Optimized structures for the AP thiophene dimer at different levels of theory are presented in Figure S2. Without dispersion corrections, DFTB methods employing either the *3ob-3-1* parameter set or our new parametrization produce structures that differ from DFT calculations, with the two molecules exhibiting minimal overlap. Despite this difference, the molecules remain positioned in parallel planes, in contrast to the T-shaped geometry observed in DFTB3/MIO calculations, as reported in the reference.[4] When D3 dispersion corrections are applied, the DFTB structures closely match the DFT reference, exhibiting a more pronounced stacking pattern with greater molecular overlap. A similar trend is observed for the caffeine sensing system (Figure S3), where the application of D3 dispersion corrections results in optimized geometries with well-stacked configurations comparable to those in the DFT reference. However, the S–S and S–N distances predicted by our new parameterizations are slightly shorter (by 0.1 to 0.3 Å) than those obtained from the DFT calculations, which can be attributed to the artificial binding effect. With respect to this, we examined the interaction energy of the H_2S dimer and the H_2S-NH_3 dimer using different DFTB methods and parametrizations. As shown in Figure S4, the *3ob-3-1* parameter set in conjunction with the DFTB3 method, significantly reduces artificial binding effects compared to the DFTB2 method using the *mio-1-1* parameter set.[2] Our new parametrizations perform better than *mio-1-1*, but not as

good as *3ob-3-1* on this property. This behavior explains the shorter S–S and S–N distances mentioned before. Notably, the smaller compression radius for the sulfur *d*-orbital used in the extended *ob2-1-1* set could solve the artificial binding issue.

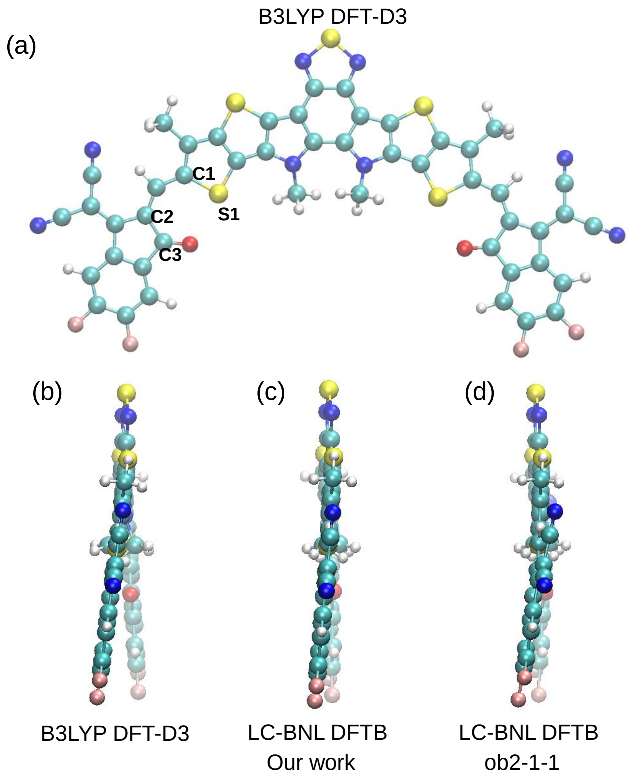

Figure S5: Optimized geometries of the acceptor material Y6 obtained using B3LYP DFT from (a) the front view and (b) the side view; and using LC-BNL DFTB from the side view with (c) a larger compression radius for sulfur used in our parametrization and (d) a smaller compression radius from the *ob2-1-1* set.

As shown in Figure S5, the dihedral angle defined by atoms S1, C1, C2, and C3 in the B3LYP DFT calculation is −1.0°, indicating a nearly planar configuration. Using our parametrization with an increased sulfur d-orbital compression radius (4.5 a_0), the LC-BNL DFTB calculation yields a dihedral angle of −0.6°, closely matching the DFT result. In contrast, when employing parameters with the same sulfur compression radius as in the *ob2-1-1* set, the dihedral angle is changed to −6.0°, deviating more significantly from planarity.

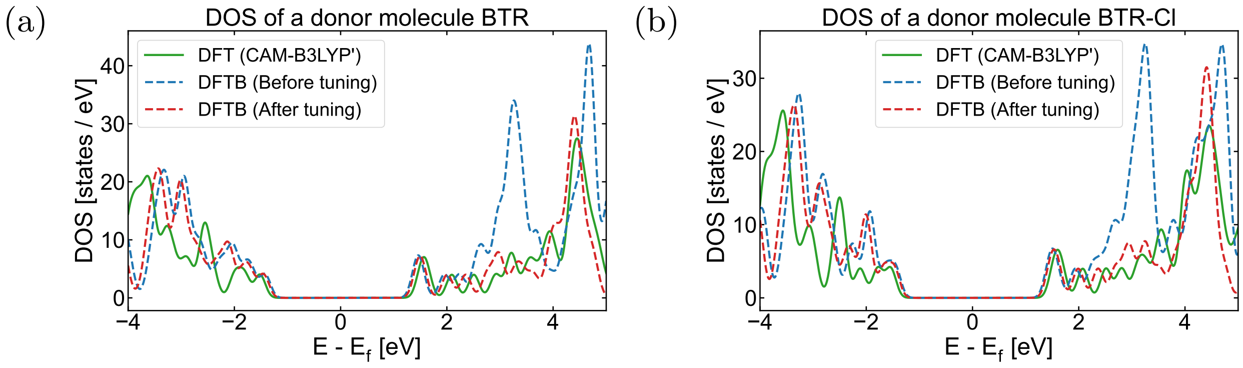

Figure S6: Comparison of the density of states (DOS) of two donor molecules (a) BTR and (b) BTR-Cl, computed on the CAM-B3LYP′ DFT and CAM-B3LYP′ DFTB levels of theory, employing $3d$-orbital on-site energies for sulfur and chlorine before and after optimization.

Table S2: D3(BJ) parameters for DFTB parametrizations using different functionals.

Parameters	s_6	s_8	a_1	a_2
D3(BJ) used in this work for all functionals	1.0000	1.4000	0.3900	5.3000
Reparametrized D3(BJ) for B3LYP	1.0000	0.6337	0.7123	2.6468
Reparametrized D3(BJ) for CAM-B3LYP′	1.0000	0.7471	0.7050	2.8327
Reparametrized D3(BJ) for LC-BNL	1.0000	0.9743	0.6879	3.2125

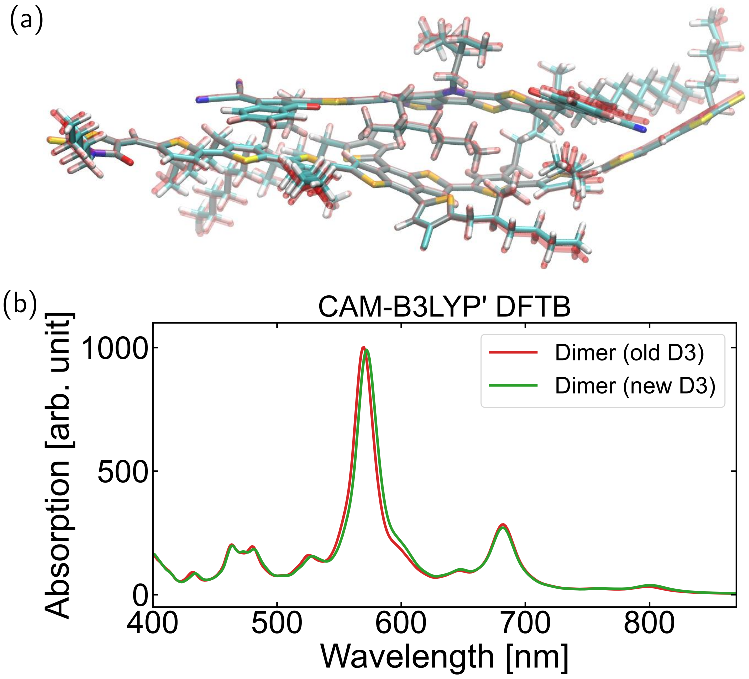

Figure S7: (a) Structural comparison of dimer geometries optimized using the old versus new D3 dispersion parameters. The colored structure represents the geometry with the new parameters, while the red shadow denotes the geometry obtained with the old parameters. (b) Comparison of the absorption spectra of dimer geometries optimized using the old versus new D3 dispersion parameters.

S2 Supporting results

HOMO-LUMO gaps

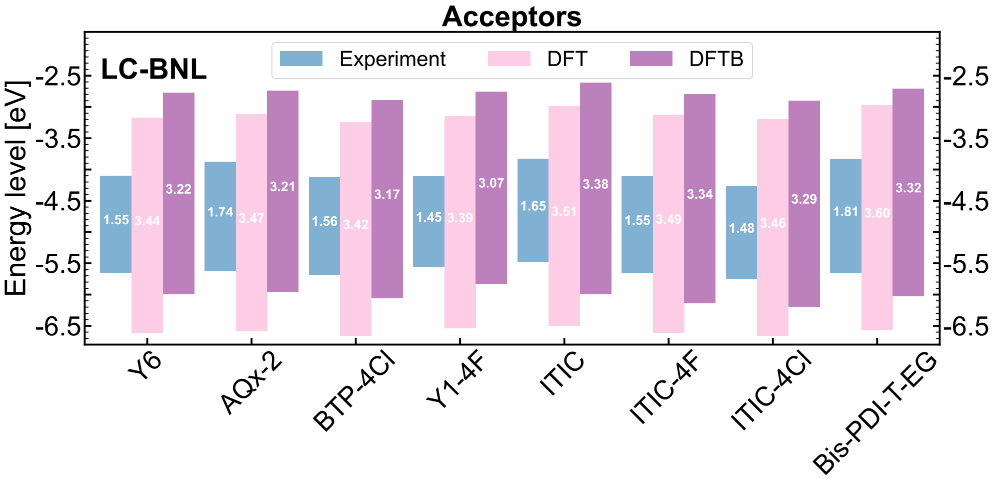

Figure S8: HOMO/ LUMO-level positions and gap sizes (in eV) of OPV acceptor molecules calculated by DFT and DFTB using LC-BNL functionals.

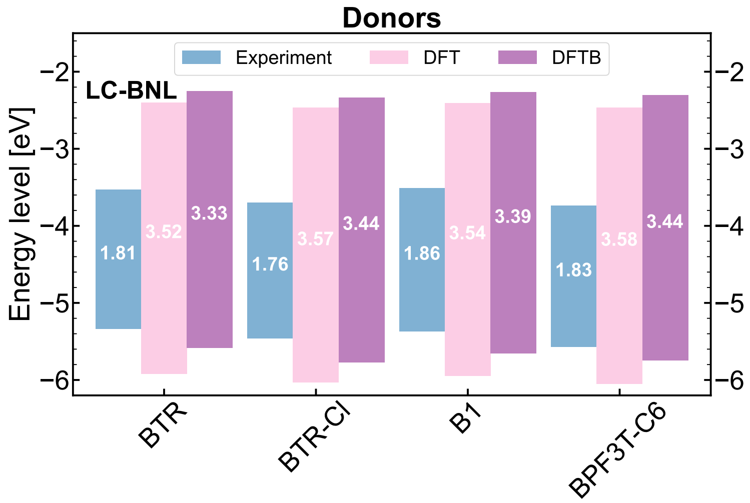

Figure S9: HOMO/ LUMO-level positions and gap sizes (in eV) of OPV donor molecules calculated by DFT and DFTB using LC-BNL functionals.

Molecular orbitals

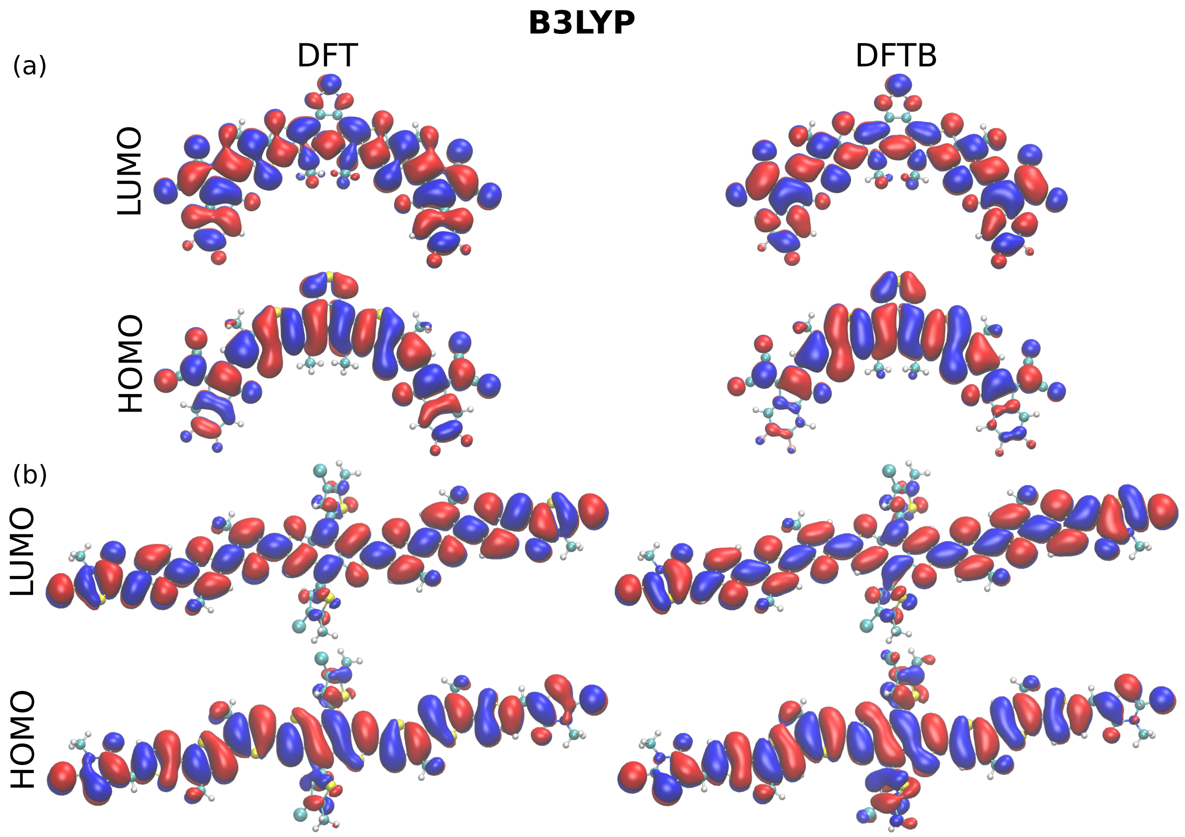

Figure S10: Visualization of the HOMO and LUMO orbitals of (a) an acceptor molecule Y6; and (b) a donor molecule BTR-Cl, using B3LYP DFT and DFTB, respectively.

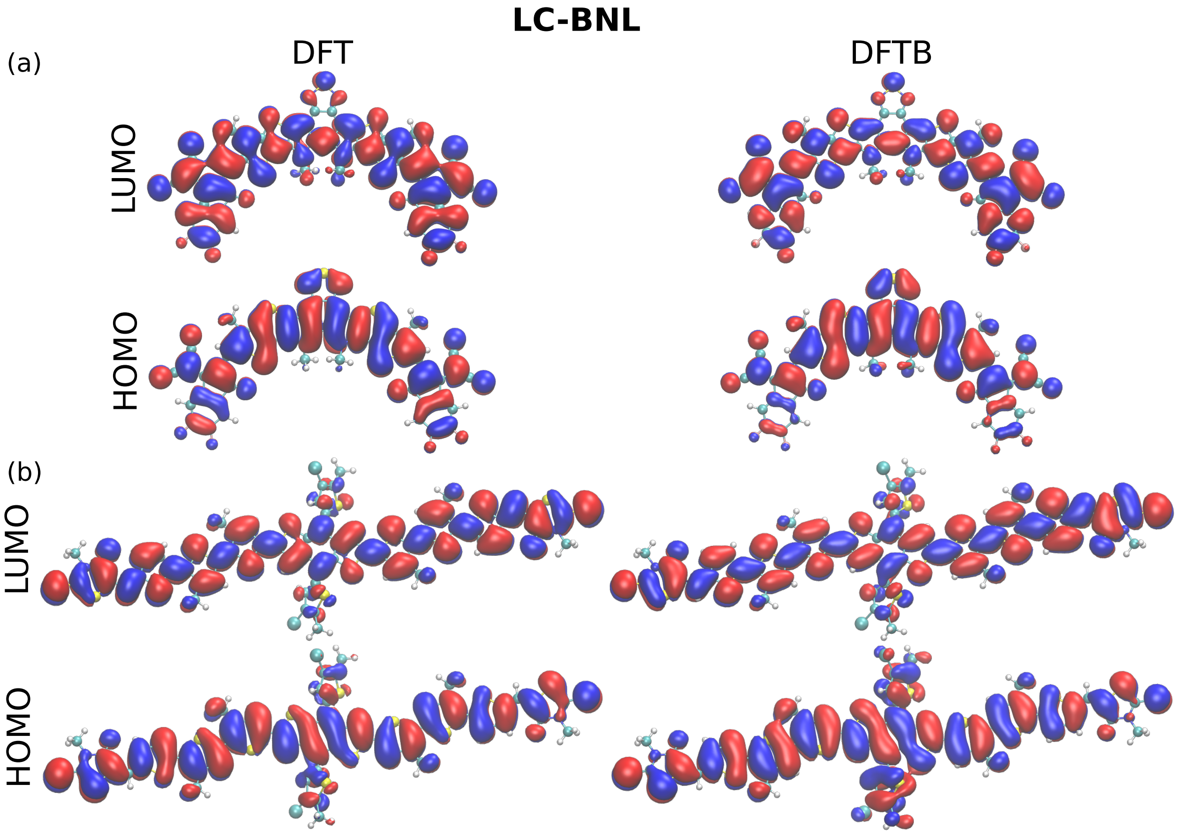

Figure S11: Visualization of the HOMO and LUMO orbitals of (a) an acceptor molecule Y6; and (b) a donor molecule BTR-Cl, using LC-BNL DFT and DFTB, respectively.

Repulsive evaluation

Table S3: Bond length of X-C (X = F, Cl, OH, SH, O, N, C) in Å.

Molecule	B3LYP DFT-D3	3ob-3-1 DFTB	B3LYP DFTB	CAM-B3LYP′ DFTB	LC-BNL DFTB
CH_2S (Thioformaldehyde)	1.618	1.615	1.609	1.614	1.602
C_2H_3SH (Vinyl mercaptan)	1.78	1.778	1.782	1.78	1.759
C_2Cl_4 (Tetrachloroethylene)	1.729	1.729	1.738	1.727	1.744
C_2H_3Cl (Vinyl chloride)	1.751	1.748	1.741	1.741	1.794
C_2F_4 (Tetrafluoroethylene)	1.324	1.343	1.359	1.366	1.338
C_2H_3F (Vinyl fluoride)	1.347	1.332	1.354	1.361	1.325
C_2H_4O (Vinyl alcohol)	1.369	1.354	1.358	1.358	1.334
CH_3OCH_3 (Dimethyl ether)	1.41	1.412	1.408	1.408	1.402
C_4H_4S (Thiophene)	1.735	1.745	1.73	1.728	1.7
C_6H_5SH (Thiophenol)	1.804	1.807	1.813	1.813	1.792
HCN (Hydrogen cyanide)	1.157	1.146	1.117	1.129	1.136
C_2H_4 (Ethylene)	1.33	1.327	1.3	1.308	1.305
C_2H_6 (Ethane)	1.529	1.514	1.532	1.534	1.507
C_6H_5F (Fluorobenzene)	1.351	1.342	1.364	1.371	1.335
$C_6H_5F_5$ (Pentafluorobenzene)	1.335	1.34	1.36	1.367	1.335

Table S4: Comparison of the average bond length (MEAN) and its standard deviation (STD) of OPV molecules in the dataset, as measured for geometries relaxed using B3LYP DFT and the B3LYP DFTB parametrization of this work. Mean absolute deviation (MAD), maximum absolute deviation (MAX), 10th and 90th percentiles of the deviation between DFT and DFTB calculated bond lengths are listed. All quantities are provided in angstrom (Å).

Bond	MEAN (DFT)	STD (DFT)	MEAN (DFTB)	STD (DFTB)	MAD	MAX	10th	90th
C−C	1.46	0.041	1.49	0.040	0.028	0.044	0.019	0.037
C=C	1.38	0.006	1.37	0.005	0.013	0.016	0.010	0.016
C≡N	1.17	0.000	1.12	0.000	0.042	0.043	0.042	0.043
C=O	1.22	0.001	1.22	0.004	0.003	0.007	0.002	0.005
C=S	1.65	0.000	1.68	0.000	0.025	0.025	0.025	0.025
C−F	1.34	0.005	1.36	0.002	0.021	0.023	0.018	0.023
C−Cl	1.74	0.004	1.74	0.002	0.003	0.004	0.003	0.004

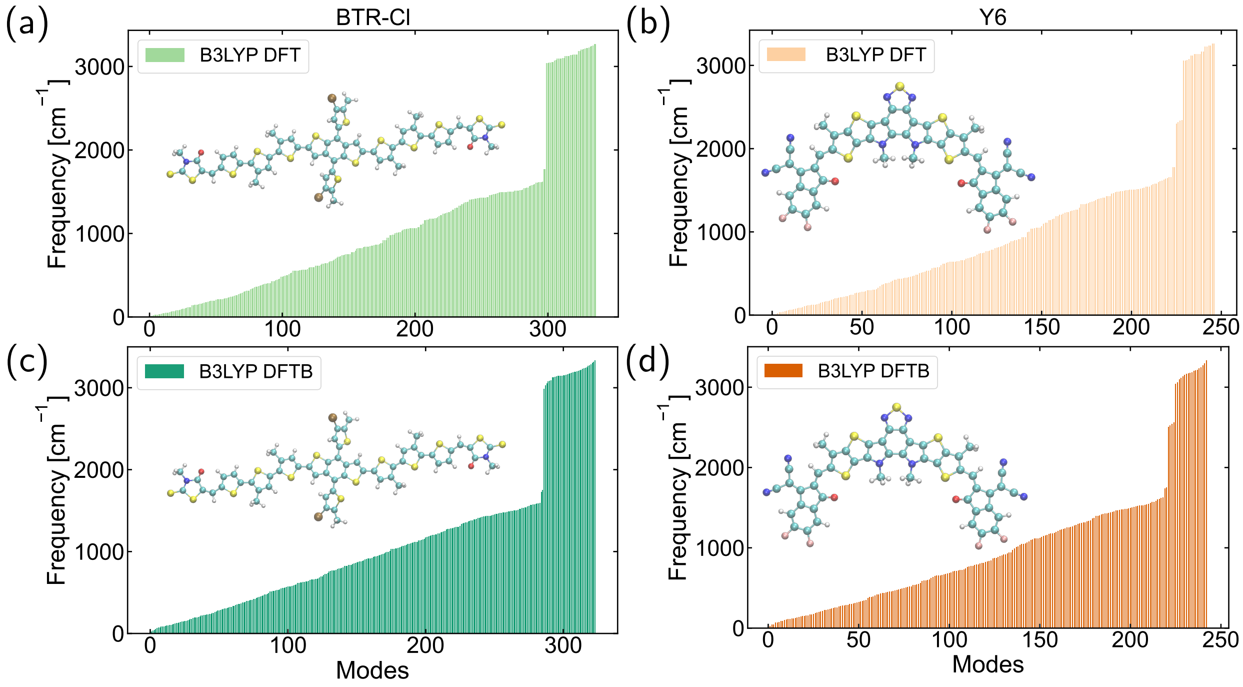

Figure S12: Comparison of the vibrational modes of the donor molecule BTR-Cl, based on (a) B3LYP DFT and (c) B3LYP DFTB; and the acceptor molecule Y6 based on (b) B3LYP DFT and (d) B3LYP DFTB (relaxed geometries shown in the inset).

Molecular dynamics

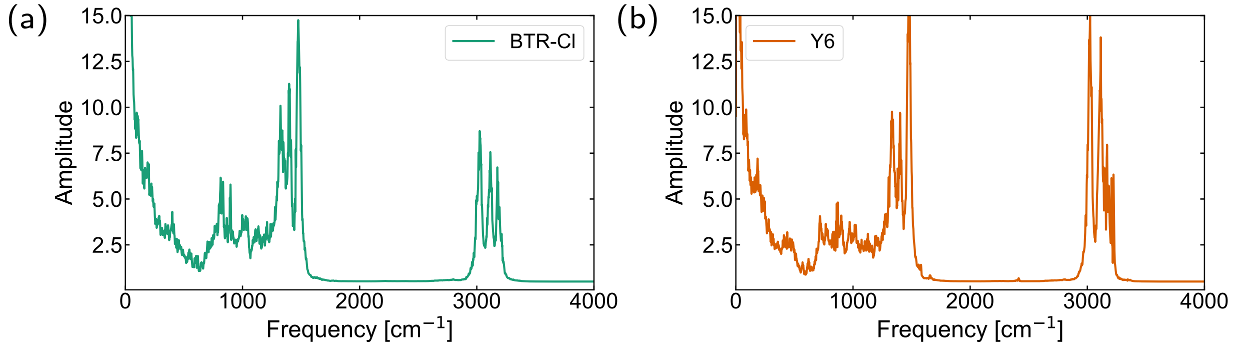

Figure S13: Anharmonic spectra from velocity autocorrelation of (a) BTR-Cl and (b) Y6, obtained by molecular dynamics simulations using CAM-B3LYP′ DFTB.

DFTB molecular dynamics (MD) simulations were performed on the individual molecules BTR-Cl and Y6, respectively. The simulations were initiated using relaxed geometries as the starting structures. A Nosé-Hoover chain thermostat was applied, with a simulation time step of 1 fs. During the MD simulations, the temperature was linearly increased from 5 K to 300 K over the first 250 steps and then maintained at 300 K for the remaining 19750 steps. Besides, the coupling strength was set to 3300 cm^{-1}. Following the simulations, velocity-velocity auto-correlation functions were computed to obtain the corresponding vibrational frequencies.

Bench-marking against other approximate DFT methods

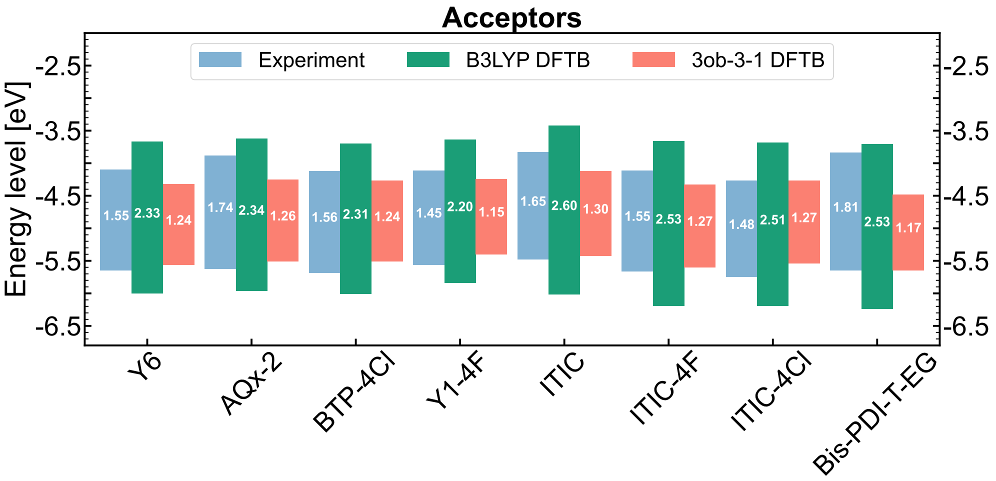

Figure S14: HOMO/ LUMO-level positions and gap sizes (in eV) of OPV acceptor molecules calculated by DFTB3 using the *3ob-3-1* parametrization against the B3LYP parametrization.

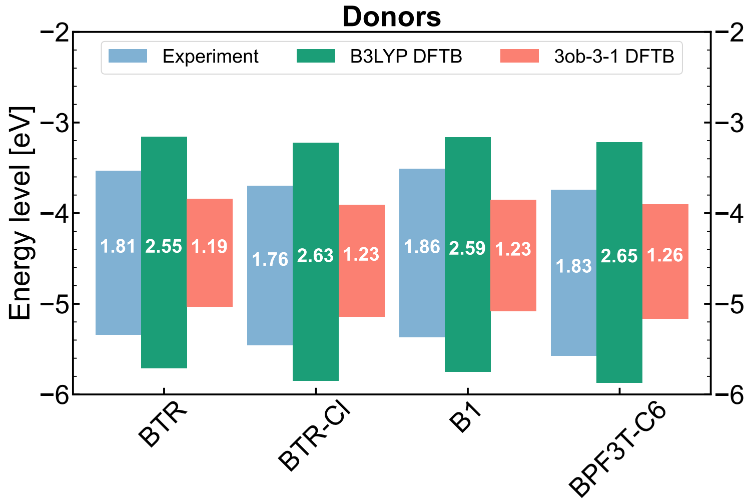

Figure S15: HOMO/ LUMO-level positions and gap sizes (in eV) of OPV donor molecules calculated by DFTB3 using the *3ob-3-1* parametrization against the B3LYP parametrization.

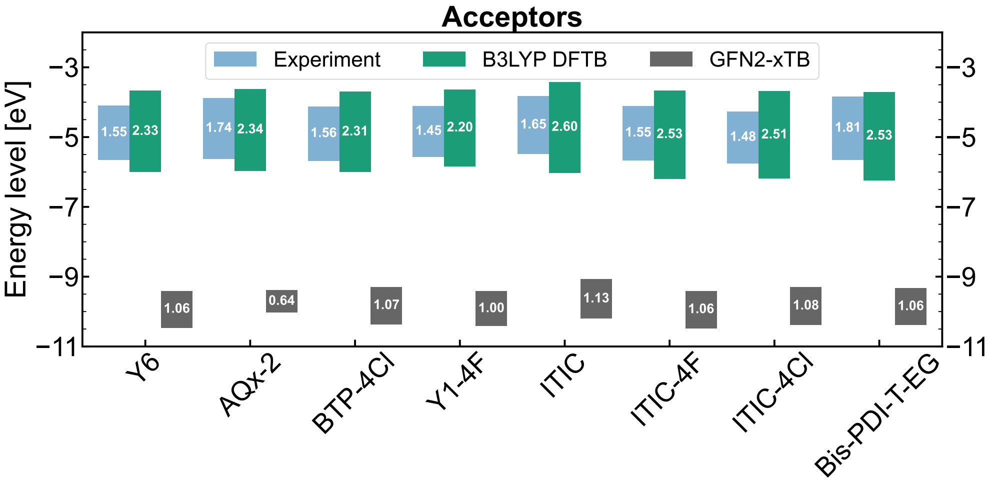

Figure S16: HOMO/ LUMO-level positions and gap sizes (in eV) of OPV acceptor molecules calculated by GFN2-xTB against DFTB using the B3LYP parametrization.

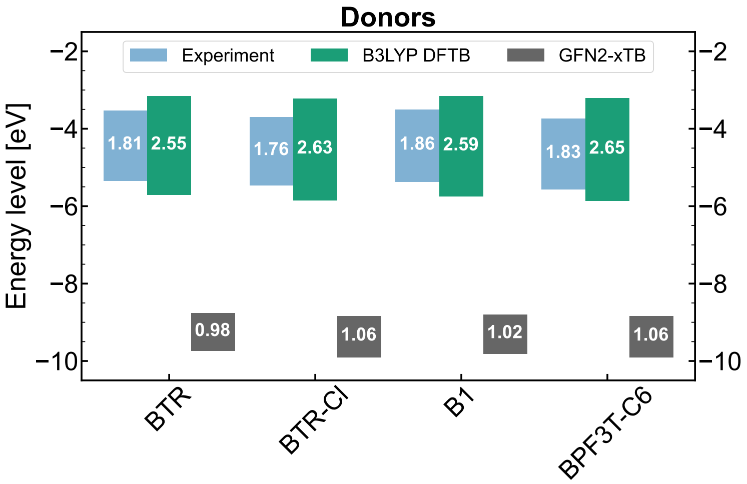

Figure S17: HOMO/ LUMO-level positions and gap sizes (in eV) of OPV donor molecules calculated by GFN2-xTB against DFTB using the B3LYP parametrization.

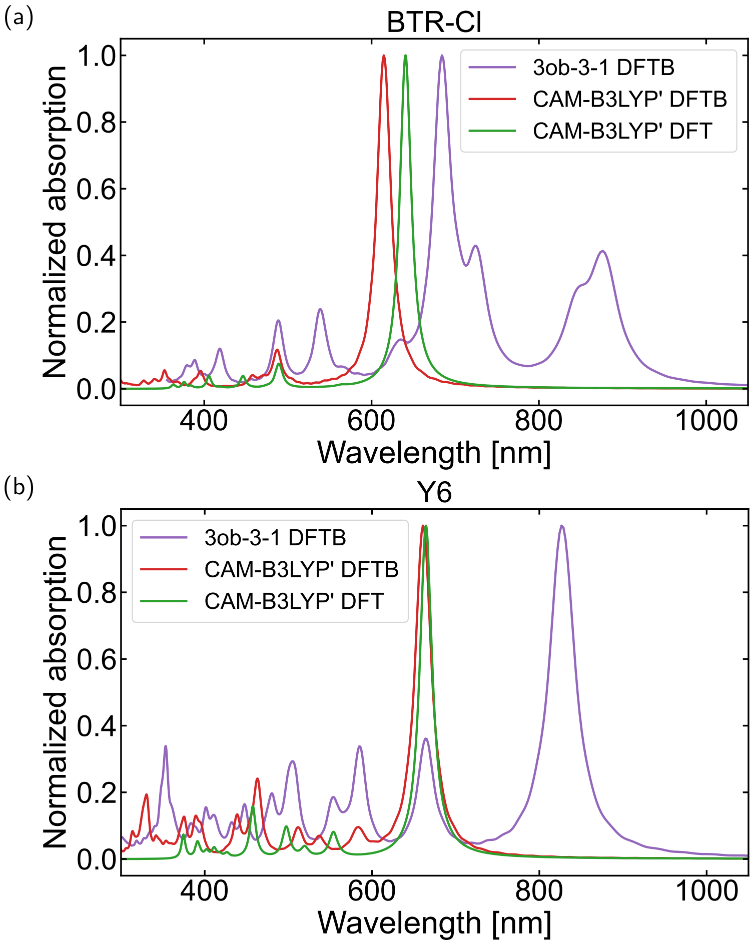

Figure S18: Comparison of normalized absorption spectra of (a) BTR-Cl and (b) Y6 single molecule calculated by CAM-B3LYP′ linear response TD-DFT, real-time TD-DFTB using the *3ob-3-1* and CAM-B3LYP′ parametrizations.

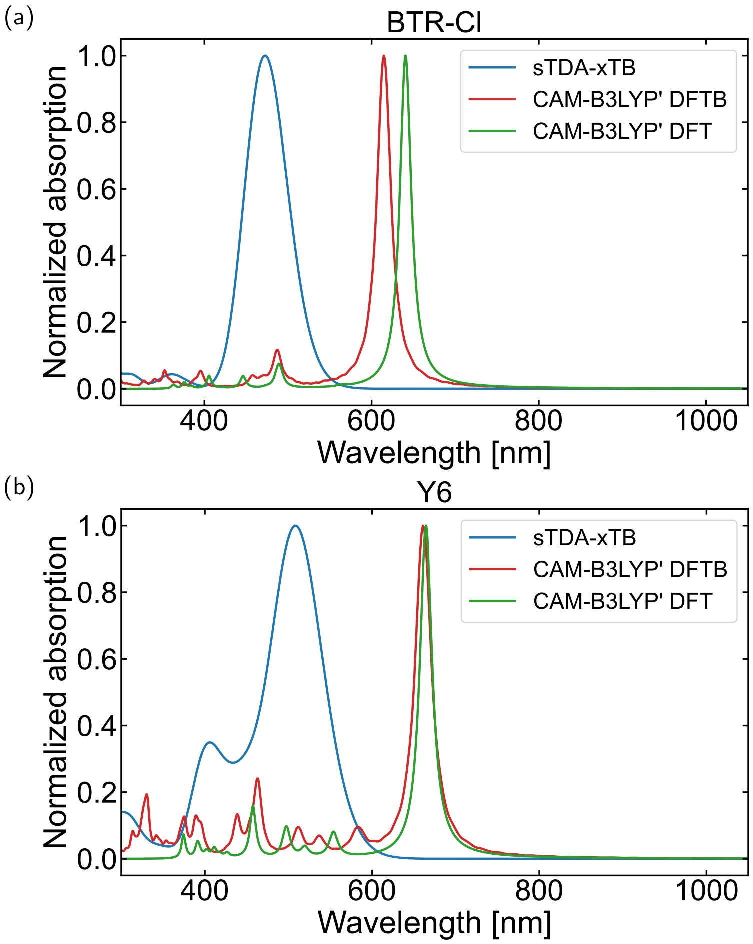

Figure S19: Comparison of normalized absorption spectra of (a) BTR-Cl and (b) Y6 single molecule calculated by CAM-B3LYP′ linear response TD-DFT, real-time TD-DFTB using the CAM-B3LYP′ parametrization, and sTDA-xTB.

Solvent effect on absorption spectrum

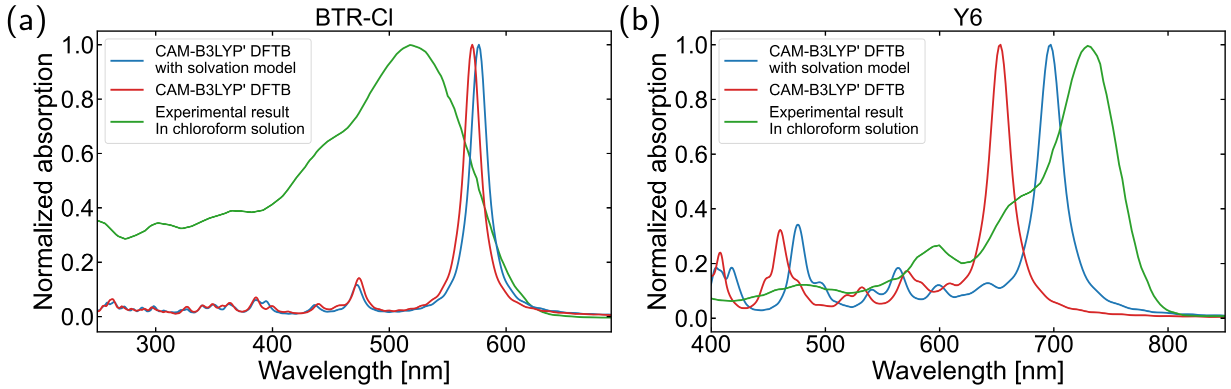

Figure S20: Comparison of the normalized absorption spectra of BTR-Cl and Y6 computed from real-time TD-DFTB simulations, both with and without a solvation model, and the experimental results for the molecules in solution (extracted from Refs. [7,8]).

Comparison of parametrizations on excited-state properties

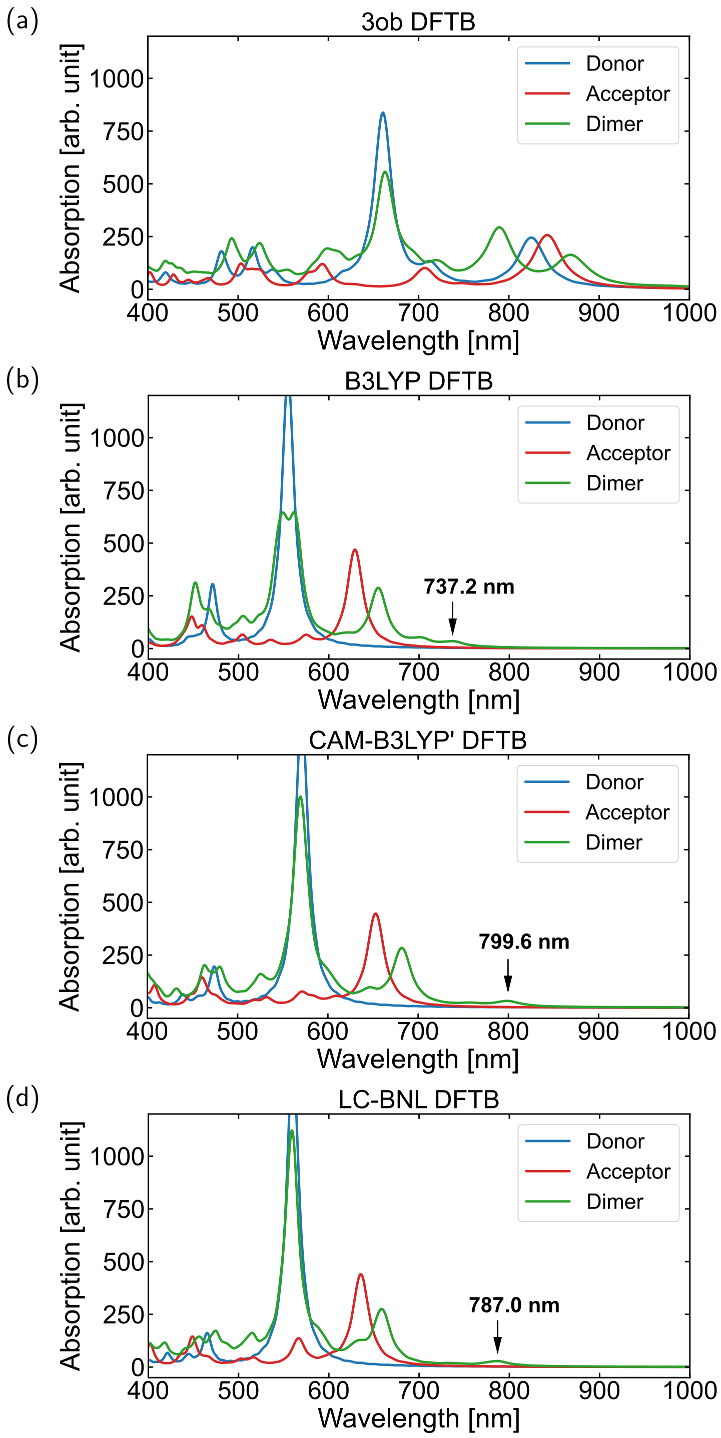

Figure S21: Absorption spectra of the isolated donor molecule BTR-Cl, the acceptor molecule Y6 (both with side-chains), and the corresponding dimer system, calculated by real-time TD-DFTB using (a) the *3ob-3-1* parameter set; (b) B3LYP; (c) CAM-B3LYP′; and (d) LC-BNL parametrization.

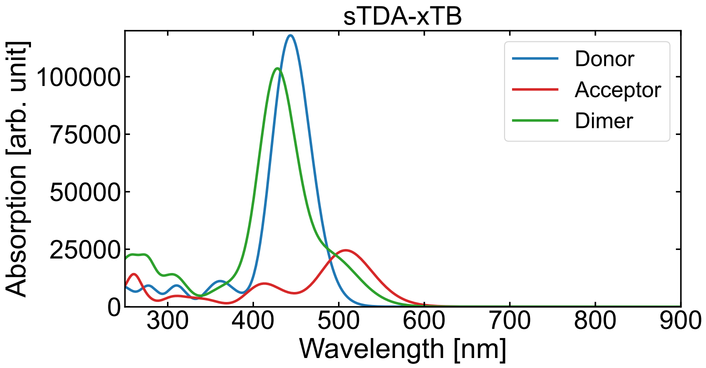

Figure S22: Absorption spectra of the isolated donor molecule BTR-Cl, the acceptor molecule Y6 (both with side-chains), and the corresponding dimer system, calculated by sTDA-xTB.

Table S5: Linear-response TD-DFTB results for the BTR-Cl and Y6 dimer system, using the B3LYP parametrization. KS denotes the dominant Kohn-Sham transition energy difference, and CT represents the charge transfer number computed using the *TheoDORE* package.

Energy [eV]	Oscillator strength	Transition	Weight	KS [eV]	CT	Symmetry
1.679	0.05989836	589 → 590	0.988	2.023	0.961	singlet
1.765	0.07892800	589 → 591	0.805	2.157	0.965	singlet
1.893	0.74691283	588 → 590	0.803	2.163	0.140	singlet
1.994	0.02772139	587 → 590	0.793	2.218	0.933	singlet
2.012	0.04824969	588 → 591	0.908	2.297	0.852	singlet
2.082	0.04854170	587 → 591	0.672	2.353	0.141	singlet
2.112	0.00368308	585 → 590	0.940	2.436	0.086	singlet
2.165	0.00346238	586 → 590	0.895	2.408	0.953	singlet
2.198	1.19283868	589 → 592	0.970	2.562	0.494	singlet
2.226	0.34567790	585 → 591	0.994	2.570	0.096	singlet
2.258	0.88021036	586 → 591	0.865	2.542	0.703	singlet
2.285	0.62509186	586 → 591	0.629	2.542	0.727	singlet
2.302	0.00025750	584 → 590	0.990	2.528	0.976	singlet
2.336	0.00987469	589 → 593	0.854	2.619	0.949	singlet
2.369	0.00000927	583 → 590	0.977	2.535	0.947	singlet

Table S6: Linear-response TD-DFTB results for the BTR-Cl and Y6 dimer system, using the CAM-B3LYP′ parametrization. KS denotes the dominant Kohn-Sham transition energy difference, and CT represents the charge transfer number computed using the *TheoDORE* package.

Energy [eV]	Oscillator strength	Transition	Weight	KS [eV]	CT	Symmetry
1.551	0.06844305	589 → 590	0.901	2.578	0.954	singlet
1.634	0.02625419	589 → 591	0.654	2.709	0.985	singlet
1.817	0.73781410	588 → 590	0.721	2.706	0.114	singlet
1.918	0.11457458	588 → 591	0.648	2.837	0.834	singlet
1.944	0.02172112	586 → 590	0.998	2.929	0.068	singlet
2.020	0.03378768	587 → 591	0.770	2.894	0.337	singlet
2.040	0.02084483	589 → 591	0.557	2.709	0.780	singlet
2.060	0.12222015	589 → 592	0.524	3.080	0.782	singlet
2.081	0.10387705	586 → 591	0.987	3.060	0.153	singlet
2.139	0.00268628	585 → 590	0.925	2.940	0.959	singlet
2.160	0.92923663	589 → 592	0.676	3.080	0.728	singlet
2.182	1.95335360	589 → 593	0.742	3.143	0.370	singlet
2.244	0.15625687	588 → 592	0.896	3.207	0.344	singlet
2.247	0.00298067	589 → 593	0.539	3.143	0.903	singlet
2.266	0.00037409	584 → 590	0.607	2.950	0.633	singlet

Table S7: Linear-response TD-DFTB results for the BTR-Cl and Y6 dimer system, using the LC-BNL parametrization. KS denotes the dominant Kohn-Sham transition energy difference, and CT represents the charge transfer number computed using the *TheoDORE* package.

Energy [eV]	Oscillator strength	Transition	Weight	KS [eV]	CT	Symmetry
1.577	0.06198702	589 → 590	0.816	2.934	0.958	singlet
1.679	0.01875418	589 → 590	0.645	2.934	0.987	singlet
1.882	0.69858333	587 → 590	0.779	3.139	0.152	singlet
1.963	0.17061422	586 → 590	0.580	3.295	0.832	singlet
2.001	0.02469869	585 → 590	0.814	3.305	0.042	singlet
2.093	0.13961555	587 → 591	0.705	3.272	0.453	singlet
2.112	0.09293025	585 → 591	0.549	3.438	0.514	singlet
2.124	0.00215092	586 → 591	0.742	3.428	0.903	singlet
2.148	0.05249241	585 → 591	0.944	3.438	0.177	singlet
2.216	2.89936320	589 → 594	0.792	3.559	0.178	singlet
2.248	0.01469835	584 → 590	0.926	3.329	0.883	singlet
2.256	0.25582603	589 → 593	0.895	3.508	0.895	singlet
2.325	0.06810645	588 → 592	0.824	3.600	0.320	singlet
2.330	0.00209575	583 → 593	0.689	3.912	0.072	singlet
2.351	0.00192590	582 → 590	0.664	3.623	0.937	singlet

Influence of side-chains on charge-transfer excitations

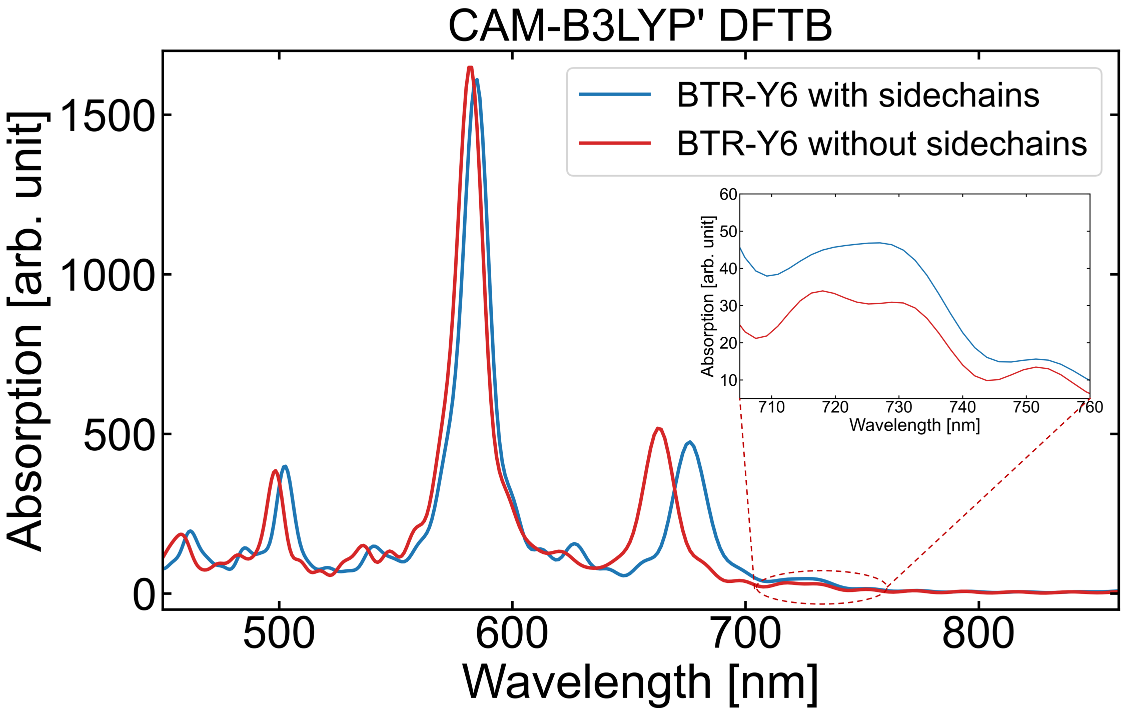

Figure S23: Comparison of the absorption spectra of the BTR and Y6 dimer system with and without side-chains. The insets display the charge-transfer excitation region on a finer scale.

DFTB MD simulation was performed on the BTR-Cl/Y6 dimer system, where the molecular cores were frozen, allowing only the atoms in the side-chains to move. The simulation was initiated using relaxed geometry as the starting structure. A Nosé-Hoover chain thermostat was applied, with a simulation time step of 1 fs. During the MD simulation, the temperature was linearly increased from 5 K to 300 K over the first 250 steps and then maintained at 300 K for the remaining 19750 steps. Besides, the coupling strength was set to 3100 cm^{-1}.

Comparison of parametrizations on charge-transfer excitations

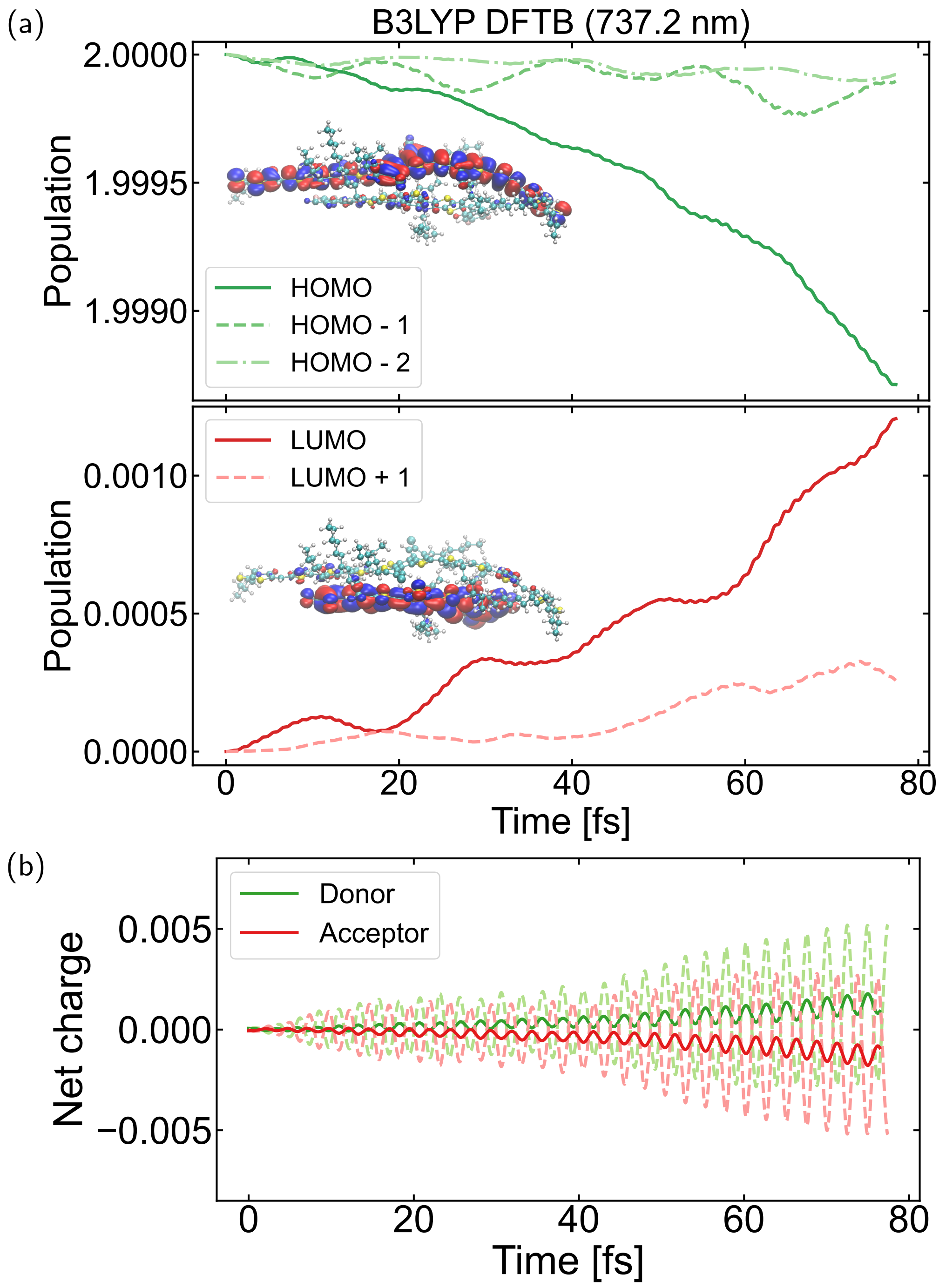

Figure S24: (a) Populations projected onto the ground state molecular orbitals, obtained by electron dynamics using B3LYP DFTB at 737.2 nm; (b) Charge-transfer between the donor and acceptor molecules during the electron dynamics simulation. Dashed lines represent the instantaneous net charge variation on each molecule as a function of time, while solid lines denote the corresponding running average.

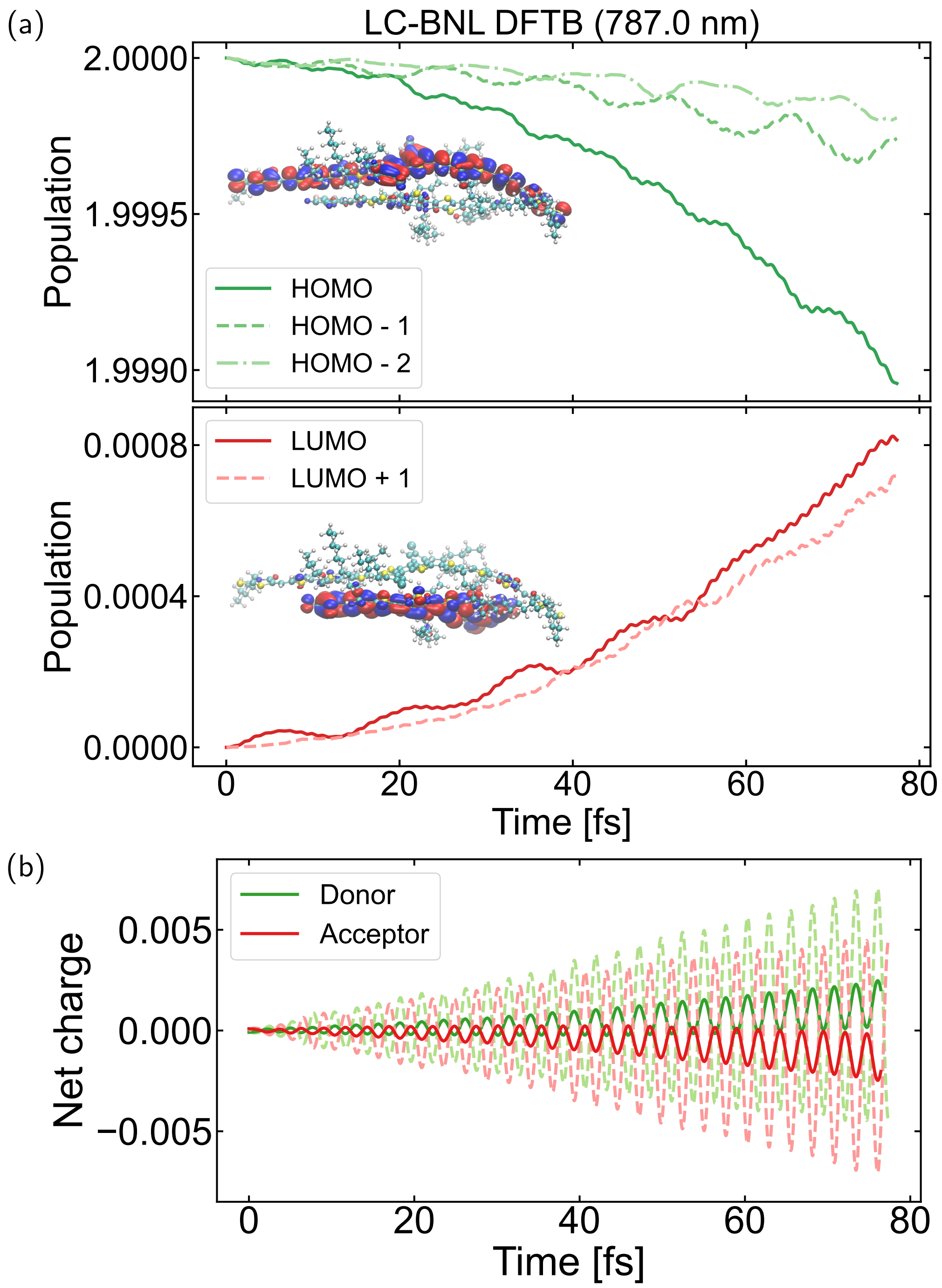

Figure S25: (a) Populations projected onto the ground state molecular orbitals, obtained by electron dynamics using LC-BNL DFTB at 787.0 nm; (b) Charge-transfer between the donor and acceptor molecules during the electron dynamics simulation. Dashed lines represent the instantaneous net charge variation on each molecule as a function of time, while solid lines denote the corresponding running average.

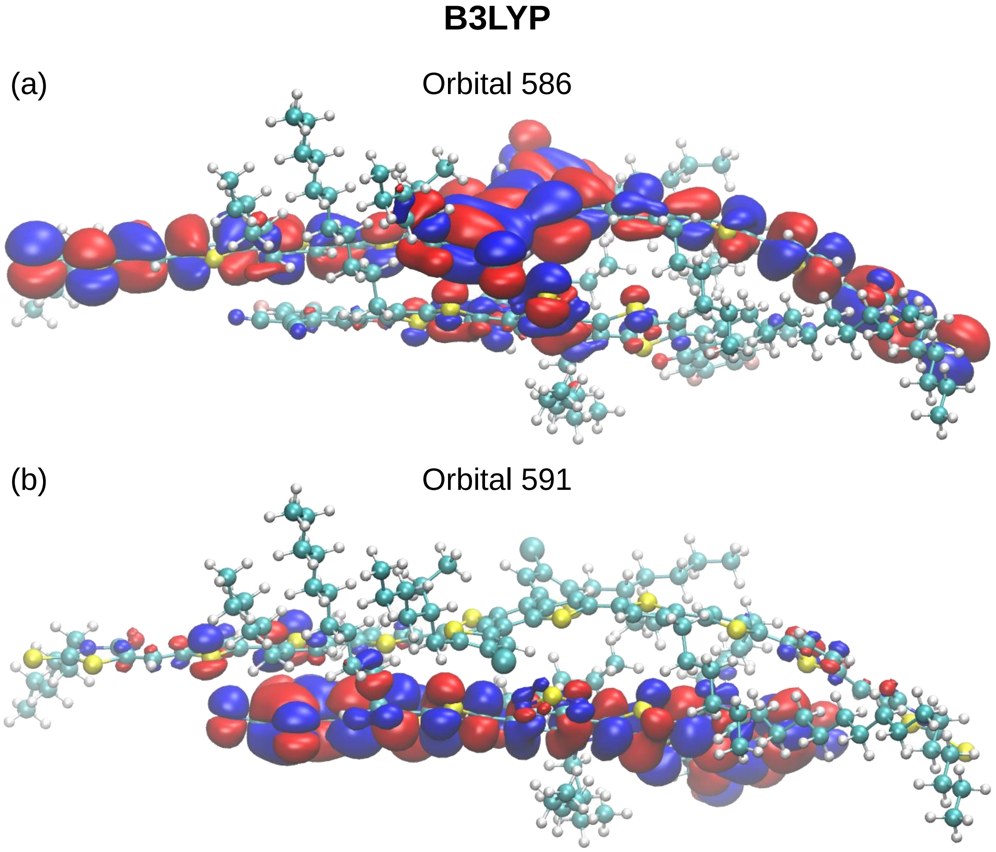

Figure S26: Visualization of the orbitals (a) 586 and (b) 591 of the BTR-Cl and Y6 dimer system, using the B3LYP parametrization. The transition from the orbital 586 to 591 contributes to the secondary peak emerging at 549 nm shown in the absorption spectra.

References

(1) Varella, M. T. d. N.; Stojanović, L.; Vuong, V. Q.; Irle, S.; Niehaus, T. A.; Barbatti, M. How the Size and Density of Charge-Transfer Excitons Depend on Heterojunction's Architecture. *J. Phys. Chem. C* **2021**, *125*, 5458–5474.

(2) Gaus, M.; Lu, X.; Elstner, M.; Cui, Q. Parameterization of DFTB3/3OB for sulfur and phosphorus for chemical and biological applications. *J. Chem. Theory Comput.* **2014**, *10*, 1518–1537.

(3) Hanwell, M. D.; Curtis, D. E.; Lonie, D. C.; Vandermeersch, T.; Zurek, E.; Hutchison, G. R. Avogadro: an advanced semantic chemical editor, visualization, and analysis platform. *J. Cheminform.* **2012**, *4*, 1–17.

(4) Petraglia, R.; Corminboeuf, C. A Caveat on SCC-DFTB and noncovalent interactions involving sulfur atoms. *J. Chem. Theory Comput.* **2013**, *9*, 3020–3025.

(5) Petraglia, R.; Steinmann, S. N.; Corminboeuf, C. A fast charge-Dependent atom-pairwise dispersion correction for DFTB3. *Int. J. Quantum Chem.* **2015**, *115*, 1265–1272.

(6) Gonthier, J. F.; Steinmann, S. N.; Roch, L.; Ruggi, A.; Luisier, N.; Severin, K.; Corminboeuf, C. π-Depletion as a criterion to predict π-stacking ability. *Chem. Comm.* **2012**, *48*, 9239–9241.

(7) Chen, H.; Hu, D.; Yang, Q.; Gao, J.; Fu, J.; Yang, K.; He, H.; Chen, S.; Kan, Z.; Duan, T., et al. All-small-molecule organic solar cells with an ordered liquid crystalline donor. *Joule* **2019**, *3*, 3034–3047.

(8) Yuan, J.; Zhang, Y.; Zhou, L.; Zhang, G.; Yip, H.-L.; Lau, T.-K.; Lu, X.; Zhu, C.; Peng, H.; Johnson, P. A.; Leclerc, M.; Cao, Y.; Ulanski, J.; Li, Y.; Zou, Y. Single-Junction Organic Solar Cell with over 15% Efficiency Using Fused-Ring Acceptor with Electron-Deficient Core. *Joule* **2019**, *3*, 1140–1151.
